# Relict high-Andean ecosystems challenge our concepts of naturalness and human impact

**DOI:** 10.1038/s41598-017-03500-7

**Published:** 2017-06-13

**Authors:** Steven P. Sylvester, Felix Heitkamp, Mitsy D. P. V. Sylvester, Hermann F. Jungkunst, Harrie J. M. Sipman, Johanna M. Toivonen, Carlos A. Gonzales Inca, Juan C. Ospina, Michael Kessler

**Affiliations:** 1 Institute of Systematic and Evolutionary Botany, University of Zurich, Zurich, Switzerland; 2 Department of Geography, Philipps-Universität Marburg, Marburg, Germany; 3 Section of Physical Geography, Faculty of Geoscience and Geography, Georg-August-Universität Göttingen, Göttingen, Germany; 4 Universidad Nacional del San Antonio Abad del Cusco, Cusco, Peru; 5 Institute of Environmental Sciences, Geoecology & Physical Geography, Universität Koblenz-Landau, Mainz, Germany; 6 Botanischer Garten und Botanisches Museum Berlin-Dahlem, Freie Universität Berlin, Berlin, Germany; 7 Department of Biology, University of Turku, Turku, Finland; 8 Department of Geography and Geology, University of Turku, Turku, Finland; 9 Instituto de Botánica Darwinion (ANCEFN-CONICET), Buenos Aires, Argentina

## Abstract

What would current ecosystems be like without the impact of mankind? This question, which is critical for ecosystem management, has long remained unanswered due to a lack of present-day data from truly undisturbed ecosystems. Using mountaineering techniques, we accessed pristine relict ecosystems in the Peruvian Andes to provide this baseline data and compared it with the surrounding accessible and disturbed landscape. We show that natural ecosystems and human impact in the high Andes are radically different from preconceived ideas. Vegetation of these ‘lost worlds’ was dominated by plant species previously unknown to science that have become extinct in nearby human-affected ecosystems. Furthermore, natural vegetation had greater plant biomass with potentially as much as ten times more forest, but lower plant diversity. Contrary to our expectations, soils showed relatively little degradation when compared within a vegetation type, but differed mainly between forest and grassland ecosystems. At the landscape level, a presumed large-scale forest reduction resulted in a nowadays more acidic soilscape with higher carbon storage, partly ameliorating carbon loss through deforestation. Human impact in the high Andes, thus, had mixed effects on biodiversity, while soils and carbon stocks would have been mainly indirectly affected through a suggested large-scale vegetation change.

## Introduction

The omnipresent effects of humans on ecosystems makes it almost impossible to properly assess past and present anthropogenic influences. Evidence is accumulating that Neolithic populations had already fundamentally changed landscapes across the globe and that, today, only completely inaccessible ecosystems remain without a direct human footprint^1, 2^. The lack of knowledge about true natural conditions^2^ is leading to a shifting baseline syndrome, with perceptions of what is “natural” becoming biased towards anthropogenically affected ecosystems^3^. Current understanding of human impact on Earth’s ecosystems stems largely from paleo-environmental proxies, but the use of such data to infer present natural ecosystem states is constrained by changed environmental conditions and because many ecosystem properties, e.g. vegetation structure and local-scale composition, cannot be readily derived from paleo-environmental proxies^4, 5^. Palynological inferences, the mainstay for baseline inferences, are further hindered by a lack of precision, with taxa only being identified to family or genus level, and an overrepresentation of taxa with windborne pollen^4^. Due to these limitations, heated debates continue over the “true” natural states of present-day ecosystems^2, 5^.

This is never more apparent than with regards to high mountain ecosystems which presently are composed largely of grassland (3.8 × 10^6^ km^2^)^6^, while paleo-ecological evidence suggests that forests once covered large proportions in the early Holocene that since declined^4, 7–14^. Some researchers argue that the drastic Holocenic forest declines can be attributed to natural climate change^9–12^, whilst others point to human impact^4, 13–16^. The challenge here is discerning causality in a situation of concurrent climate change, human population expansion, and forest decline. In the Andes, humans are known to have been present above 4,000 m a.s.l. since as far back as 12,800 years ago^17^ but the ecological impact of these early inhabitants remains unknown. Interestingly, palynological studies report a high abundance of *Polylepis* forest in the high Andes prior to the appearance of humans, but which suffered a drastic decline around 11,000 years ago^7, 8, 14^, which some researchers attribute to human activity^14, 16^. However, it is disputed whether primitive hunters, early livestock farmers or nomads, likely to have been present in small numbers, were capable of causing this large-scale forest decline and whether it was purely a result of natural causes^10^.

When considering natural ecosystems, most would say they have more species, are more fertile, and store more carbon. Actually, grazing may increase local plant species richness by altering competitive interactions^18^ while conversion of forest to grassland can increase soil organic carbon (SOC) stocks^19^. These insights, however, stem from grazing exclosures, which allow assessment of ecosystem recovery but not of natural conditions^20^.

Here we use inaccessible relicts of natural vegetation in the Peruvian high Andes, located close to human habitation and associated ecosystem-shaping disturbances of grazing and burning, to infer modern baselines and gain an understanding of human impact on these ecosystems. We consider sites that are completely inaccessible to anthropogenic disturbance to host pristine vegetation that is certain to have never received direct human impact and is representative of the potential natural vegetation based on current ecological conditions of our study area. Evidence of natural grazers (i.e. mountain deer, *Hippocamelus antisensis* d’Orbigny and viscacha, *Lagidium peruanum* Meyen) and isolated natural fires (i.e. trees damaged by lightening) were found at these inaccessible sites and they can, thus, be considered representative of natural ecosystem processes. Much controversy and debate centre around similar research from lowland Amazonia^21–23^ that attempt to infer natural vegetation from sites which “found little to no evidence of either human occupation or forest/landscape modification”^23^ as it is argued that these sites could have been accessed by humans. As our study sites have clearly always been isolated from possible human impact by steep rock walls that can only be climbed with mountaineering equipment, we can confidently say that the data we present is from ecosystems with no direct human impact.

**Figure 1:**
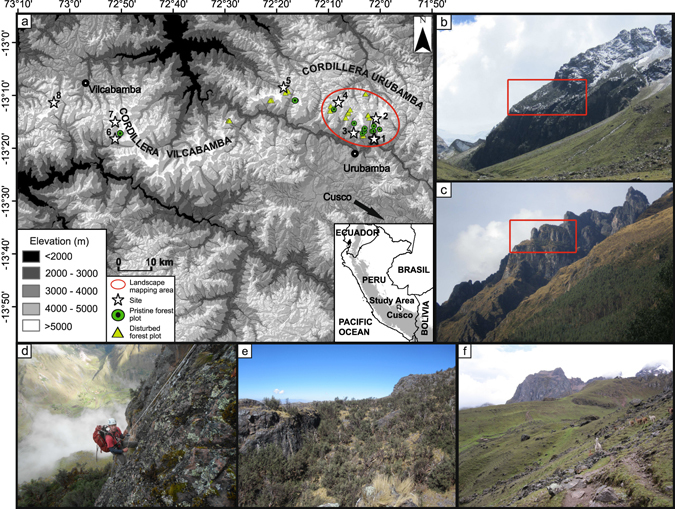
Map and impression of the study sites. (**a**) Map of the study area showing the eight study sites (denoted by numbered stars), the locations of inaccessible and accessible forest plots (denoted by green-filled circles and yellow-filled triangles, respectively), and the area used for landscape mapping of woodland and grassland in accessible and inaccessible areas (denoted by a red oval). (**b**,**c**) Example**s** of study site**s** with the inaccessible ledge**s** studied circled in red with the accessible human-impacted grasslands in the foreground. (**d**) first author abseiling into an inaccessible site. (**e**) Example of an inaccessible site with open forest intermixed with grassland. (**f**) Example of an accessible site. Photographs (**b**,**c**,**e**,**f**) taken by S.P. Sylvester; (**d**) taken by F. Heitkamp. ASTER Global Digital Elevation Model (GDEM) v2 data raster map presented in (**a**) is a product of NASA and METI and is distributed by NASA LP DAAC (https://lpdaac.usgs.gov/). Map generated using ArcGIS v.10.1 (http://www.esri.com/).

We compare processes associated to two different spatial extents: habitat conversion (forest to grassland) at the landscape scale, and habitat alteration (within a vegetation type) at the site scale. In 2010, we located undisturbed, natural vegetation in the Cordilleras Urubamba and Vilcabamba close to the ancient Incan capital Cusco, a centre of both human cultural development and crop diversity (Fig. 1). These relict ecosystems were found on extensive mountain ledges only accessible using mountaineering equipment and thus without livestock, timber extraction, or human-induced ground fires (Figs 1 and S1). They were slightly inclined and large enough (c.0.2–1.5 km^2^) to support the development of deep soils and zonal slope vegetation that does not represent specialized ecosystems linked to rock faces, and were comparable to the surrounding human-impacted landscape in all state factors^24^ and most abiotic variables. In exploratory studies, we were able to validate the proposition that these mountain ledges can be used to infer potential natural vegetation^25^ and soil development^26^ in comparison to anthropogenically shaped landscapes and ecosystems found in close proximity.

## Results

### Landscape vegetation mapping

**Figure 2:**
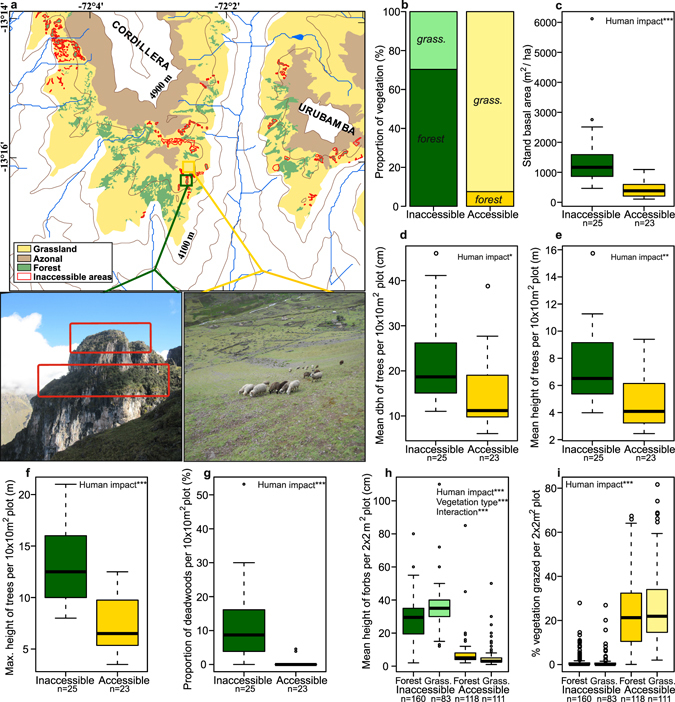
Comparisons of inaccessible and accessible vegetation structure. (**a**,**b**) Example of the landscape mapping approach showing an inaccessible and nearby accessible site (**a**) and the total proportion of forest and grassland of zonal vegetation in the Cordillera Urubamba between 4200 m and 4900 m calculated from this (**b**). (**c**–**g**) Comparisons of forest structure i.e. stand basal area (**c**), mean dbh (**d**), and mean (**e**) and maximum height (**f**) of trees, and proportion of deadwoods (**g**), per 10 × 10 m^2^ forest plot in accessible and inaccessible *Polylepis* forests. (**h**,**i)** Comparison of mean height of herbaceous vegetation (**e**) and vegetation cover grazed (**i**) per 2 × 2 m^2^ plot in inaccessible and accessible forest and grassland. Significant relationships between the structural properties and fixed effects (human impact; vegetation type) found upon analysis using generalized linear mixed models (GLMM’s) are noted within the figures (***p < 0.001, **p < 0.01, *p < 0.05; Tables S13 and S14). Photographs by S.P. Sylvester. Map generated using ArcGIS v.10.1 (http://www.esri.com/).

Only 1.1% of the Cordillera Urubamba was occupied by inaccessible zonal vegetation, while 56.9% was accessible zonal vegetation, with the remaining 42.0% corresponding to azonal habitats such as landslides, rock faces, glacial moraines, stream margins, and bogs. Inaccessible zonal landscapes had 70% forest cover whereas accessible landscapes only had 7.5% forest cover (Fig. 2a,b and Table S1).

### Vegetation assessment

**Figure 3:**
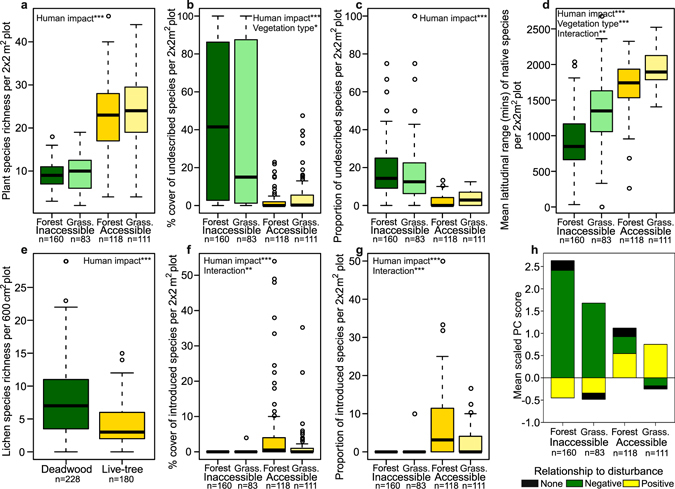
Plant diversity, composition and traits in inaccessible and accessible forest and grassland. (**a**–**h**) Comparisons of species richness of vascular plants per plot (**a**), cover and proportion of undescribed (**b**,**c**) and introduced (**f**,**g**) plant species per plot, and average latitudinal amplitude of native plant species per plot excluding introduced species (**d**). Epiphytic lichen species richness (**e**) is compared between deadwood substrate, generally only found in inaccessible forests (Fig. 2g), and live trees. To compare disturbance-related plant traits (**h**), the cumulative means of scaled principal components, referring to positive and negative disturbance related plant trait syndromes, averaged over all species per 2 × 2 m^2^ plot, are compared (Fig. S3 and Table S6). Significant relationships between the structural properties and fixed effects, found upon analysis using GLMM’s, are noted within the plots (***p < 0.001, **p < 0.01, *p < 0.05; Tables S15 and S16).

When comparing vegetation between accessible and inaccessible habitats, inaccessible forests, dominated by species of *Polylepis* Ruiz & Pav., contained higher tree stem density, with larger trees, and more standing deadwood (Fig. 2c–g) as well as denser and taller herbaceous vegetation with signs of grazing by natural herbivores (Figs 2h,i and S2a–c). Due to dominance of a few species, inaccessible habitats had about 1/3 of the vascular plant alpha diversity of accessible habitats (Fig. 3a). Most importantly, the dominant species in the herbaceous vegetation of inaccessible habitats were mostly species new to science^27–30^ (Fig. 2b,c and Table S2) or species with restricted range sizes (Figs 3d and S2d). Inaccessible forests were particularly important as hosts for these ‘new’ species, with 25% of species highlighted as indicators for this vegetation type being undescribed^27–29^ (Tables S2–S4). Inaccessible forests also had a unique and highly diverse (Fig. 3e) epiphytic lichen flora specific to the abundant standing deadwood, with indicator species analyses retrieving 28 indicator species for deadwoods but none for live trees (Table S5). Sites accessible to humans had a greater proportion and cover (Fig. 3f,g) of introduced alien plant species, which were nearly absent in inaccessible sites. Indicator species analyses found accessible forest to harbour a high proportion of these aliens (Table S3) while a few undescribed species^31, 32^ were also found to be significant indicator species of accessible grassland (Tables S3 and S4). Widespread generalist species (Figs 3d and S2d and Table S4), most of which had adaptations to burning and grazing (Figs 3h and S3 and Table S6), dominated accessible sites.

### Soil assessment

**Figure 4:**
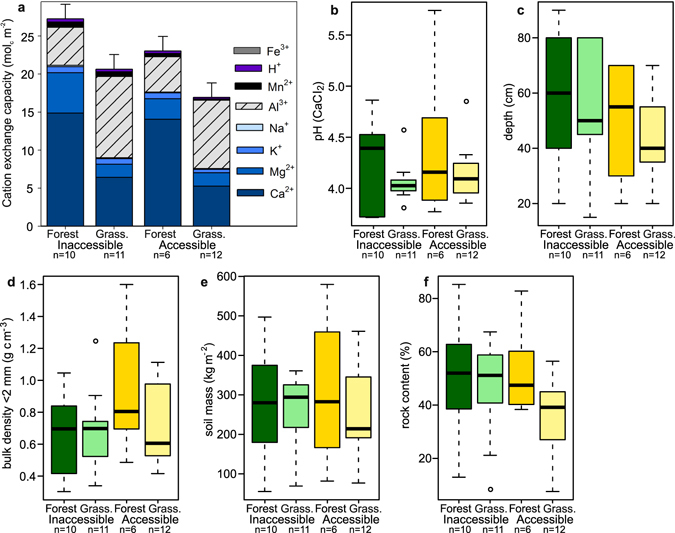
Comparisons of soil properties between inaccessible and accessible forest and grassland. (**a**–**f**) Comparisons of exchangeable cations (**a**) and pH-values (**b**) as indicators for nutrient availability in soils, and differences in depth (**c**), bulk density (**d**), mass (**e**), and rock content (**f**) of soils. Analysis with linear mixed models found no significant relationship with human impact for any of the properties apart from Na^+^, whilst Al^3+^, K^+^, Ca^2+^, Mg^2+^ had a significant relationship with vegetation type (Table S17).

Vegetation type was found to strongly influence soil properties. Most forest soils were classified as Phaeozemes (9 of 10 on inaccessible sites, 5 of 6 on accessible sites), whereas grassland soils were mostly more acidic Umbrisols (7 of 11 on inaccessible sites, 10 of 12 on accessible sites; Table S7). However, we did not find significant differences in soil properties when comparing inaccessible and accessible sites within a vegetation type (Fig. 4).

All soils were characterised by having a thick A-horizon, overlying continuous rock. The shallowest profiles were located at site 1 and were only 15 cm (inaccessible grassland) and 20 cm (inaccessible forest) deep. These soils were classified as Leptosols. They had a mollic horizon (≥0.6% soil organic carbon, Munsell colour and chroma ≤3, ≥0.6% more organic carbon than parent material, a base saturation ≥50% and ≥10 cm thickness overlying continuous rock) and were classified as Mollic Leptosols. All other soils were deeper than 25 cm and had less than 80% (by volume) rocks. All soils fulfilled the criteria of colour and carbon concentration to be classified as either Phaeozeme or Umbrisol, the difference being the low (>50%) base saturation in Umbrisols (Table S7).

Soil depth ranged from 15 to 90 cm with the median between 40 and 60 cm without significant differences between habitats (Fig. 4c). Bulk density showed surprisingly high values in grazed forests (Fig. 4d). This may be explained by the tendency of livestock to rest under the shelter of the canopy. Compaction was not obvious in accessible grassland for the whole profile, but bulk density was higher in the top 10 to 20 cm. Overall, the mass of fine soil did not differ between habitats (Fig. 4e), indicating that erosion was not higher in grazed habitats.

### Carbon stock assessment

**Figure 5:**
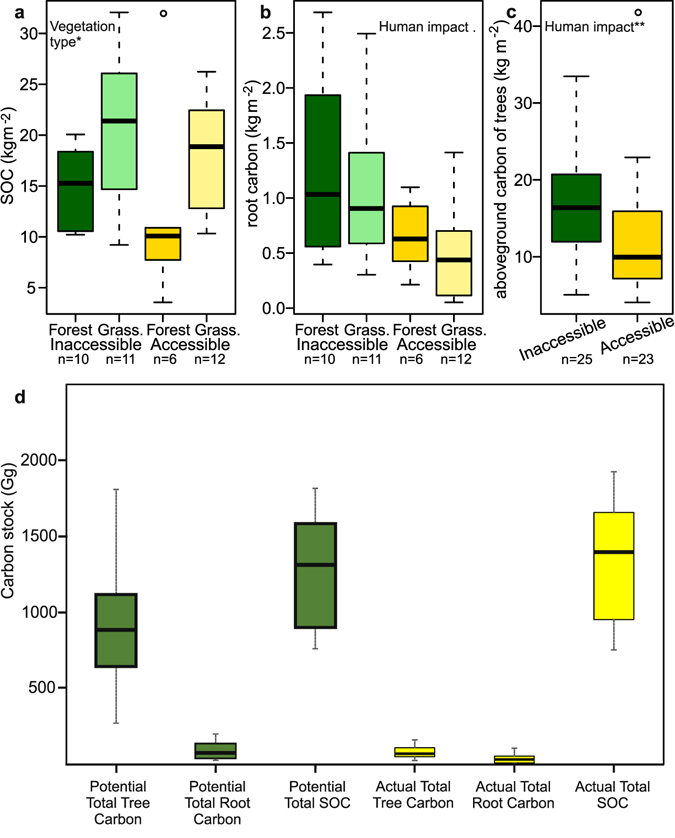
Comparisons of carbon properties between inaccessible and accessible forest and grassland (**a**–**c**) and comparison of potential and actual landscape carbon stocks (**d**). (**a**–**c**) Comparisons of carbon contents in Soil Organic Carbon (SOC) (**a**), root biomass (**b**) and aboveground biomass of trees (**c**). Significant relationships between the structural properties and fixed effects, found upon analysis using GLMM’s, are noted within the figures (**p < 0.01, *p < 0.05, p < 0.1; Table S18). (**d**) Estimated values of the potential (green) and current (yellow) carbon stocks for aboveground tree carbon, root carbon and soil organic carbon (SOC) at the landscape scale for the high elevation area mapped of the Cordillera Urubamba. Values estimated using carbon stock values from inaccessible and accessible forest and grassland (Table S8) extrapolated over the areas calculated using landscape mapping (132,660 km^2^; Tables S1 and S9). Actual values of the carbon stocks can be found in Table S9.

Soils held the largest carbon stocks, followed by tree carbon, with root carbon being relatively insignificant (Fig. 5a–c and Table S8). At the site scale, no significant impact was noted for soil or root carbon but for above-ground tree carbon, accessible forests had on average 25% lower stocks than inaccessible forests (Fig. 5a–c and Table S8). At a landscape scale, assuming that the inaccessible sites are representative of the natural variability in the landscape and that zonal areas would hypothetically contain 70% forest, transformation from forest to grassland is predicted to have decreased tree and root carbon stocks by roughly 90% and 60%, respectively, but increased soil levels by 5% (Fig. 5d and Table S9). These counteracting effects imply that total carbon stocks may have decreased by about 35% at the landscape scale (Tables S9 and S10).

Our root biomass estimates (mean 1.8 kg m^−2^; 0.1–5.4 kg m^−2^) are much higher than those of Oliveras *et al*.^33^ (0.6 kg m^−2^ for puna grassland at 3500 m) and Hertel & Wesche^34^ (0.2 kg m^−2^ for *Polylepis* woodland at 4000 m). These studies, however, reported sampling depths of 30 cm and 2 cm mineral soil, respectively. Taking these differences in sampling depth into account, our data fit quite well.

## Discussion

Even though our findings on human influence on vegetation agree, to some extent, with former ideas^4, 14–16, 18^, the degree to which humans have affected high Andean vegetation is unprecedented. The almost complete replacement of the highly endemic, previously undescribed, plant species dominating natural vegetation by “weedy” species adapted to grazing and burning is a finding that would have been impossible to predict in the absence of baseline data. This study also shows us how human impact is degrading high Andean forests through giving us a first glimpse of what potential natural forest structure should look like, with denser forests, larger trees and abundant deadwood that harbour a unique signature of cryptogam diversity, something that has not been recognised until now. Deadwoods are known to be important for biodiversity conservation in other parts of the globe^35^ and we show here that they enhance cryptogam diversity in Andean forest ecosystems. They are also likely to have other benefits, e.g., for invertebrates and birds, of which *Polylepis* forests harbour a high number of endemic and threatened species^36^.

As inaccessible habitats are dominated by forest but disturbed accessible landscapes have very little (Fig. 2b), this supports the notion^14, 16^ that human activity is the main cause of the current dominance of grassland and that, in the absence of humans, a tree-dominated forest-grassland mosaic would be found over large proportions of the high Andes. This pristine forest-grassland mosaic encountered in our study sites supports the idea that high Andean forests would not be continuous across the landscape in the absence of people, as evident from the palaeoecological record^10, 12^. As accessible areas harbour c.10% of the forest cover found in inaccessible areas (Fig. 2b), we could assume that c.90% of forest cover has been lost, although this estimate is drawn from the static assumption that pristine ecosystem properties are the same as those of the inaccessible sites studied here, while the true scenario is likely to be more dynamic.

Considering the large changes in vegetation, one may expect concomitant changes in soil properties. Contrary to our expectations, we found no significant differences in soil properties when comparing inaccessible and accessible sites within a vegetation type. Our results, thus, contrast with general preconceptions^37^ that pastoral practices lead to a depletion of available nutrients in the soilscape, with local enrichment at resting or watering places^38, 39^. We did, however, find a strong influence of vegetation type, with the differences between soils being mainly connected to nutrient availability, in this case exchangeable cations. Grassland vegetation appears to have low cation demand^40^, lower evapotranspiration, and less interception of precipitation^41, 42^ compared with forests. Consequently, this may cause higher leaching rates leading to a depletion of Ca^2+^ and Mg^2+^, whilst less mobile cations, such as Al^3+^, are relatively accumulated^26^. High levels of organic matter (Fig. 5a) usually reduce the effect of Al through complexation: While organic matter in soils can bind to many different substances, it is especially reactive towards the complexation of cations in general and, in particular, to multi-charged cations like Al^3+^ and Fe^3+^ ^43^. As all the soils studied had a high organic matter content, and thus a strong binding to Al remaining in the organic matter matrix of the soil, Al^3+^ is therefore relatively accumulated in grassland because the less charged cations are more easily leached^26^. This organic matter matrix also buffers the pH lowering effect of Al and explains why there were no significant differences in pH between the different soil types. Forest vegetation, on the other hand, functions as a “nutrient pump” with tight cycling of the most important nutrients^44^ and can experience nutrient replenishment through interception of ion rich cloud water^45^. In our case, this effect was reflected in the exchangeable cations Ca^2+^ and Mg^2+^ (Fig. 4a) that were more abundant in forest.

Erosion was also not higher in disturbed habitats, as one might expect. Most frequently, anthropogenic pressure on steep land induces erosion with an estimated global value of 50–200 Pg of soil being lost each year^46^. Nevertheless, the high livestock densities at the study sites did not lead to any obvious signs of erosion, indicating a sustainable land use. This may be explained by how, in the entire region, paths of livestock cover the slopes (Figs 1f and S1h,i), which serve as small terraces breaking the high relief energy. Moreover, sheep and camelids have soft hooves, which do not wound the soil surface. Another possible explanation may relate to how high contents of organic matter (Fig. 5a), a primary bonding agent in aggregation^47^, may protect soils against erosion.

As soils are mainly influenced by the vegetation type, the large-scale deforestation suggested by our landscape mapping study may have also led to a large-scale change in the soilscape, favouring acidic umbrisols. At a landscape scale, the natural acidification process during soil development appears to be accelerated by man-induced vegetation change because, although there were no significant changes in pH, the greater abundance of exchangeable Al^3+^ indicates that the proton buffer, which is aluminium dissolution at these pH-values (Fig. 4b), is already more depleted in grassland soils. Proton buffering is a major non-linear ecological function and soils enter whole new process domains once the threshold of a certain buffer is surpassed, with consequent acidification bursts^48^. These sudden drops in pH found in grasslands affect transformation and translocation (biogeochemical) processes^49^. Consequently, vegetation-soil feedbacks^50^ are expected to be much stronger in man-made landscapes where grasslands dominate.

Pulling the vegetation and soil components together and looking at carbon stocks, we found that human influence had fundamentally different impacts depending on the scale studied. At the site scale, human impact was seen to have no effect on SOC and relatively little effect on root carbon, but a large impact on aboveground tree carbon. However, when looking at overall carbon stocks at the landscape scale, and if we assume a hypothetical 90% forest reduction as calculated in the landscape mapping study, we see that human impact has led to only a 35% decrease of carbon stocks. This is due to soils of grassland, the dominant vegetation type in human-impacted landscapes, holding disproportionately large amounts of SOC compared to forest soils. Thus, despite a suspected massive decline in forest cover (Fig. 2b), carbon stocks can still be seen to be relatively little affected which is a novel notion for the study of human impact on ecosystem carbon stocks^19, 20^.

The hypothetical landscape values calculated here, and reconstructions of the potential natural vegetation and soils of these high Andean landscapes, still remain uncertain as not all ecotopes in the landscape are present on the inaccessible ledge sites studied by us and certain factors, such as hydrology, were not tested. Furthermore, while we assume that inaccessible sites are subject to natural fire frequencies and grazing densities, based on our finding evidence of natural fires and grazing (Fig. 2i), there will be variability in these natural ecosystem processes across the landscape which we may not have accounted for. Information on natural grazing densities and fire frequency is still lacking or incomplete from these high elevation environments. Nevertheless, taruka (mountain deer) and viscacha, the main natural grazers of the Cordilleras Urubamba and Vilcabamba, were abundant in the inaccessible sites (*S*.*P*. *Sylvester pers*. *observation*). The other natural South American camelid grazers, guanaco (*Lama guanicoe* Müller) and vicuña (*Vicugna vicugna* Molina), are not known from the study area but, even if present, would have grazed largely on the valley floors or very high-elevation grasslands^51^, which were not covered in this study. Our findings of similar pristine vegetation types in all pristine sites studied, regardless of varying topography and climate, and observations of the low impact of natural grazers on pristine vegetation, support the notion that the vegetation found in these inaccessible sites is representative of the potential natural vegetation of our study area. Further research, covering all variability in the landscape, would allow us to refine these reconstructions of potential natural vegetation and estimations of human impact further, but this would depend on locating extensive pristine landscapes, which probably no longer exist.

Overall, our study highlights how seemingly moderate human influence by extensive pastoralism and associated burning of grasslands can cause both tremendous and unpredictable shifts in ecosystems properties. Forests would, apparently, be more widespread in these landscapes, but would not cover the entire area, while previously undescribed species, today very rare or extinct on grazed accessible areas, would dominate the herbaceous layer of the landscape. Despite these major changes in vegetation, the estimated decrease in total ecosystem carbon stocks was less than expected in relation to the estimated loss of forest cover. Soils were also surprisingly stable against anthropogenic pressure when compared within vegetation types, and even accumulated carbon. Nevertheless, their sensitivity to vegetation change is suggested to have created a landscape dominated by a more acidic soil type, irreversibly reducing the capacity to buffer future acid inputs into the system. Our data do not allow inferences over whether the pristine vegetation documented in this study could be representative of the widespread natural vegetation found after the last glacial period nor during an earlier interglacial period. However, by identifying critical taxa to differentiate natural versus human-affected vegetation types, it sets the stage for palynological studies to address this question.

More generally, considering the simultaneous changes in climate and human immigration to the Andes in the early Holocene, our study poses the question whether vegetation development after the last glaciation was natural or human-influenced right from the beginning. The notion that the high elevation grasslands of the Andes are a largely man-made or, at least, man-maintained environment has far-reaching implications for our understanding of human and ecosystem development in the Americas as well as for future land management and conservation.

## Methods

### Location and site conditions

The study was conducted in the Cordillera Vilcabamba and the Cordillera Urubamba of the Cusco region, in the southern Peruvian Andes (13°08′–13°15′S, 72°18′W, 4200–4900 m a.s.l.) over 4 years between 2010–2014 (Figs 1 and S1). The vegetation is classed as ‘puna’ and is characterised by extensive tracts of tussock grassland interspersed with patches of *Polylepis* Ruiz & Pav. (Rosaceae) forest. *Polylepis* forests in the Cordillera Urubamba and Vilcabamba contain a single species of *Polylepis* and, often, another *Gynoxys* Cass. (Asteraceae) species per forest. The Cordillera Urubamba had forests that were dominated by either *Polylepis racemosa* or *P*. *subsericans*, and with *Gynoxys nitida* Muschl. often occurring as a shrub in inaccessible plots. The Cordillera Vilcabamba had forests that were dominated by *Polylepis pepei*, with inaccessible plots also having large trees of *Gynoxys cuzcoensis* Cuatrec. as an important component of forest structure. The vegetation of the sites contained elements of flora characteristic of both the humid Páramo, found in northern Peru^52, 53^, and the dry Puna, which occupies the majority of the high elevation landscapes of central and southern Peru^54–56^.

All sites were characterised by low annual mean temperatures, a high diurnal temperature amplitude (Table S11), and a pronounced dry season from May to October. The Cordillera Vilcabamba sites were generally more humid than the Cordillera Urubamba sites due to receiving updrafts of humid air from the Amazon basin. Site 5, located at a mountain pass facing the Cordillera Vilcabamba (Fig. 1a), receives a large amount of humidity from updrafts of moist air passing across the pass and, thus, exhibits a vegetation and climate more characteristic of the Cordillera Vilcabamba. This difference in humidity is reflected by the recordings of mean annual precipitation in the closest climate stations which range from 454 mm in the Cordillera Urubamba (Urubamba, 2863 m a.s.l.) to 1606 mm in the Cordillera Vilcabamba (Winaywayna, 2800 m a.s.l.)^16^. Since our study sites lie >c.1400 m above the climate stations, the data cannot be used to infer precipitation regimes at our study sites. Nevertheless, orography and vegetation pattern in the areas studied indicate that mean annual precipitation ranges at the higher end of these records. *In situ* records of air relative humidity and temperature for each Cordillera, averaging all datalogger sources (DS1923 Hygrochron iButtons), indicate that the Cordillera Urubamba sites generally experienced greater temperature variation, with colder extremes, and air humidity was lower (Table S11).

The accessible vegetation of the sites is subject to annual or biennial burning to remove undesirable tree saplings and old unpalatable tussock grasses, and to promote the resprouting of palatable new shoots. Animal trampling was widespread on accessible slopes, as typical for the whole region. Trampling resulted in a kind of small-scale terracing without destroying vegetation cover and, hence, erosion was only triggered near settlements but not on a large scale. Studies indicate that pre-Incan civilizations were present in the Cusco region for, at least, 4000 years^57–60^ and it is likely that these sites have been intensively used as rangeland during this time.

### Landscape vegetation mapping

Spatial distribution of grassland and forest in anthropogenically accessible and inaccessible areas of the Cordillera Urubamba was mapped based on high resolution aerial photographs and Landsat 8 satellite images with ArcGIS over an area of 132,660 km^2^ (Figs 1a and 2a), from the upper limit of crop cultivation at 4200 m to the uppermost forest patches at 4900 m. We used a Landsat 8 satellite image based normalized difference vegetation index to extract areas without vegetation. Due to the coarseness of Landsat data, we also manually mapped azonal areas (landslides, moraine, water bodies). Valley bottoms above 4200 m were excluded based on topographic information (ASTER Global DEM Version 2 of 30 m). Grassland cover was calculated by subtracting the cover data of forests and azonal areas. Inaccessible zonal areas were manually mapped based on the criteria that they were surrounded by steep (>80°) rocky terrain.

### Field design

Vegetation was categorized into four habitat types: accessible forest, accessible grassland, inaccessible forest, inaccessible grassland, across sites spanning the Cordilleras Urubamba and Vilcabamba (Figs 1 and S1). 8 large study sites were chosen (Fig. 1a) where all vegetation and soil attributes were studied from multiple plots within the four habitat types. Plots of grassland vegetation were located in open areas at a distance >10 m from the closest tree. 10 × 10 m^2^ forest plots were studied from the 8 main sites as well as many other inaccessible and accessible areas throughout the Cordillera Urubamba and Vilcabamba (Fig. 1a).

Inaccessible sites were selected based on being (a) completely inaccessible to human disturbance (i.e. the spread of human induced groundfires, livestock grazing and trampling, and human disturbances such as firewood harvesting) and (b) hosting zonal vegetation comparable in most abiotic variables (e.g., climate, geological parent material, topography, elevation, aspect, degree of soil and vegetation development) to accessible sites found adjoining them. Inaccessible sites were found on large ledges ranging in size from c.0.2–1.5 km^2^ (Fig. 1b,c), with deep soils (soil depth ranged from 15 to 90 cm with the median between 50 and 60 cm; Fig. 4c). These sites were found alongside accessible slope sites, with all sites located close (1–3 km) to human habitation and the associated disturbances of grazing, burning, firewood harvesting etc. Inaccessible sites differed from accessible slope sites in being surrounded by cliffs that inhibited access to domesticated livestock and the spread of human-induced ground fires with our access made possible through the use of modern mountaineering equipment and knowledge. For sites 1–8 (Fig. 1a), accessible vegetation plots and soil profiles were located as close as possible to the inaccessible vegetation plots and soil profiles, with the horizontal distance no more than ca. 500 m within any particular site. Climate did not differ significantly between inaccessible and accessible areas (Table S12). Natural grazing animals, taruka (mountain deer; *Hippocamelus antisensis* d’Orbigny) and viscacha (*Lagidium peruanum* Meyen), were present at all inaccessible sites based on sightings, tracks, and scat.

### Vegetation assessment

Above-ground forest structure was assessed by comparing height and dbh (diameter at breast height; 130 cm) of all phanerophytes, whether live or standing deadwood, with a stem circumference greater than 10 cm at breast height (130 cm) from 48 10 × 10 m^2^ plots in inaccessible and accessible forests spread across the Cordilleras Urubamba and Vilcabamba (Fig. 1a). Plots were situated within the forest stands at a distance of at least 20 m from the forest edge to minimize edge effects. Inaccessible forest plots were composed of 5 plots of *Polylepis pepei* B.B. Simpson forest, 7 plots of *P*. *racemosa* Ruiz & Pav. forest, and 13 plots of *P*. *subsericans* J.F. Macbr. forest. Accessible forest plots were composed of 8 plots of *Polylepis pepei* forest, 6 plots of *P*. *racemosa* forest, 3 plots of *P*. *sericea* Wedd. forest and 6 plots of *P*. *subsericans* forest.

Stand basal area was calculated for each forest plot by, firstly, converting dbh to tree basal area for every tree present in each 10 × 10 m^2^ forest plot using the equation:

1 $$BA=\frac{\pi \times {(DBH/2)}^{2}}{144}$$

These values of tree basal area were then summed up for each plot and divided by the area to give the stand basal area in m^2^ ha^−1^ for each plot.

Vascular plant diversity, composition, and herbaceous vegetation structure were assessed by comparing 472 2 × 2 m^2^ plots from the four habitat types at sites 1–8 (Fig. 1), with 10–33 2 × 2 m^2^ plots being studied from representative patches of vegetation within each habitat type for each site. At sites 1 and 5, the ‘accessible forest’ habitat type was not present due to extensive deforestation in the area^25, 26^ and so plots were made in the other three habitat types. Plots were spaced with at least 5 m distance between them to sample as much variability in the vegetation as possible. Within each 2 × 2 m^2^ plot, percentage cover of each vascular plant species, percentage of vegetation showing signs of grazing, cover of bare soil and litter, was estimated by overlaying a grid on the plot. The average height of forbs was measured from soil level, averaging values of 10 measurements. Maximum height was measured from the tallest forb present in the plot. A total of 334 vascular plant species were recorded from all plots. Voucher specimens were collected and identified in the field or in the herbarium and are deposited at CUZ, LPB, USM, Z. Help in identification was given by Dr. Nicholas Hind (Asteraceae), Dr. Ulf Mollau (*Bartsia*), Dr. Zulma Rugulo (*Calamagrostis*), Dr. Xenia Villavicencio (*Calamagrostis*), Simon Pfanzelt (*Gentianella*), Dr. Michael Sundue (Grammitideae), Dr. Klaus Bernhard von Hagen (*Halenia*), Hibert Huaylla (Iridaceae), Dr. Colin Hughes (*Lupinus*), Dr. Robert Soreng (*Poa*), Dr. Paul Peterson (Poaceae), Dr. Simon Laegaard (Poaceae), Dr. Fred Barrie (*Valeriana*). Nomenclature of vascular plants follows W^3^ TROPICOS (http://www.tropicos.org/).

Life-form and grazing-related morphological traits were scored for all species of vascular plant encountered in the plots following Diaz *et al*.^61^ and Miehe *et al*.^62^. The 32 traits scored can be found in Table S6. Species lifeform was categorized based on Raunkiær’s lifeform classification^63^. As the majority of tree species measured between 3–12 m, we combined the classes Mesophanerophyte and Microphanerophyte. Arboreal vascular hemiparasite *Tristerix longebracteatus* (Desr.) Barlow & Wiens was included in the epiphyte category whilst grassland vascular hemiparasites in the genera *Bartsia* L., *Calceolaria* L. and *Castilleja* Mutis ex L. f. were classed as hemicryptophytes.

Latitudinal range size (difference between the northern- and the southern-most specimen record) was calculated for 324 vascular plant species encountered in the plots using specimen and distribution data from W^3^ TROPICOS (http://www.tropicos.org/) and literature references therein. Latitudinal range size was considered a good proxy for overall range size as the majority of widespread species were distributed throughout the length of the Andes. This approach has been employed in other studies of Andean vegetation^64–66^. Values were recorded at the grain size of latitudinal minutes. To be conservative, when distribution data by country were used, the latitudinal limit was taken from the most southern or most northern alpine area of that country.

Environmental variables (elevation, average topsoil depth before first rock hit, stoniness of soil, wind exposure, slope inclination, aspect) were recorded for each plot. Average soil depth before first rock hit was measured in 10 different parts of each plot using a metal rod driven into the ground until detained by the first rock. Stoniness of the soil was semiquantitatively measured based on the soil depth measurements and given a categorical value of 0–100. Wind exposure was given a categorical value based on the author’s opinion of the relative exposure of each plot to wind. Slope inclination was measured using a clinometer with slope exposition being recorded using a compass. Soil depth was calculated for sites 1–4 from soil profiles (3 per habitat type) dug to the C horizon with the length of the whole profile being measured (see ‘Soil sampling’ below). Air relative humidity and temperature were recorded at 7 of the 8 sites (sites 1–6 and 8; Fig. 1a) using digital dataloggers (DS1923 Hygrochron iButtons). Two dataloggers were used per site, one datalogger being placed in the accessible forest habitat (or placed under bunch grasses at sites 1 and 5) and one in the inaccessible forest habitat type. Dataloggers were placed on raised platforms 10 cm from the soil surface and were sheltered from direct insolation. They recorded at 2 hourly intervals over a 12-month period. Daily mean, daily mean minimum and daily mean maximum, and absolute minimum and maximum air temperatures and relative humidity were calculated from the data collected (Table S11) and compared (Table S12).

Differences in epiphytic lichen diversity and composition between living and standing deadwood *Polylepis* trees was assessed following Gradstein *et al*.^67^. In the inaccessible forest habitat type of sites 1, 2, and 4, four live and four deadwood trees were studied and, at site 3, three live and three deadwood trees were studied, resulting in a total of 30 trees. Sites 1 and 3 had *P*. *subsericans* forest, while sites 2 and 4 had *P*. *racemosa* forest. Trees were located at a minimum distance of 20 m from the forest edge to minimise edge effects, were spaced at least 20 m apart, and had a similar height (6–7 m) and dbh (c. 20 cm). 20 × 30 cm^2^ plots exhibiting a representative cryptogam diversity were studied from three areas of each tree: bole of 0–2 m, the inner canopy and the last 60 cm of branches, with four samples from each cardinal direction being collected for each area^67, 68^. Thus, a total of 408 plots, 16 plots in each living tree and 12 plots in each dead tree, were evaluated. The samples were preserved by air drying. Voucher specimens of the lichens can be found in CUZ, B, LPB, Z. Nomenclature of lichens follows that of Index Fungorum (http://www.indexfungorum.org).

### Soil assessment

Soil parameters were assessed for the four habitat types from sites 1–4 of the Cordillera Urubamba with three pits per habitat type resulting in a total of 39 soil profiles. Relief and parent material differed slightly between sites, which was included into the structure of the statistical model using “site” as a random effect. All sites in the Cordillera Urubamba were located well above the shoulders of the U-shaped valleys, indicating no direct glaciation in the last glacial maximum. Positions of the pits were chosen to avoid major accumulation or erosion processes and were proximate to vegetation plots. Pits were dug by hand as deep as possible, reaching down to continuous bedrock. After recognising that soils comprised only one, sometimes very thick, A-horizon, sampling was done in depth increments (0–5, 5–10, 10–20, 20–30, 30–40, and >40 cm). Volumetric samples were taken from each layer using the volume replacement method^69^.

For soil mass calculation, moist field samples were scaled and sieved to 2 mm. The rock and organic residue on the sieve was cleaned with tap water and separated. Roots were dried at 70 °C, rocks at 105 °C and both were scaled after cooling in a desiccator. Subsamples of the fine earth were dried at 40 °C and 105 °C (48 hours) for analysis and determination of dry mass^70^. Calculations of the mass of fine earth per square meter were done as follows^69^:

2 $${\rho }_{w,i}=\frac{{W}_{fe,i}+{W}_{r,i}}{{V}_{i}}$$

3 $${M}_{w,i}={\rho }_{w,i}\times {h}_{i}$$

4 $${M}_{fe}=\sum _{i=1}^{n}\frac{{M}_{w,i}\times 100}{({W}_{r,i}/{W}_{fe,i}\times 100)+100}$$

where *ρ*_*w*_ is the bulk density of the weathered pedon (i.e., fine earth and rock fragments; g cm^−3^) of layer *i*, *W*_*fe*,*i*_ is the weight of oven-dry fine earth (g) of layer *i*, *W*_*r*,*i*_ is the weight of rocks (g) in layer *i*, *V*_*i*_ is the total volume (g cm^−3^) of the undisturbed sample (i.e., the hole in the profile wall) in layer *i*, *M*_*w*,*i*_ is the mass of the weathered pedon (kg m^−2^) in layer *i*, *h*_*i*_ is the height (mm) of layer *i* and *M*_*fe*_ is the mass of fine earth (kg m^−2^) of the whole profile. The bulk density of fine earth (*ρ*_*fe*_, g cm^−3^) of layer *i* was calculated as:

5 $${\rho }_{fe,i}=\frac{{W}_{fe,i}}{{V}_{i}-({W}_{r,i}/2.65)}$$

Where 2.65 is the assumed density of rocks (g cm^−3^).

To calculate root biomass, a subsample of soil (20 g) was soaked in demineralized water and poured over a 250 µm sieve^71^. Remaining soil particles and roots were divided by swirling and decantation and handpicking with tweezers. Roots were dried (70 °C) and scaled and the total biomass was calculated based on the proportion of the subsample compared with the total sample and the soil mass. Since roots were sampled in order to have an estimate of this carbon stock, we made no distinction according to species and did not separate dead from living roots.

Carbon and nitrogen were measured by dry combustion at 950 °C (Truspec CHN LECO, St Joseph, MI, USA). Carbon and nitrogen concentrations were converted to stocks based on the soil mass calculated. Low pH values (measured in CaCl_2_) indicated that soil carbon did not contain carbonates. Due to the low pH-values, we assumed that total carbon equals soil organic carbon^72^. Measurements of pH were done in a slurry with 1:5 (v/v) soil-to-solution (0.1 m CaCl_2_) ratio (ISO 10390:2005). Exchangeable cations were extracted to indicate nutrient availability at actual soil pH. Effective cation exchange capacity was determined by the extraction procedure of Lüer & Böhmer^73^ in un-buffered solution. Results, therefore, represent the cation exchange capacity, i.e. nutrient availability, at actual soil pH. Exchangeable aluminum, iron, manganese and hydrogen is also called “exchangeable acidity” and aluminum can be toxic to plants. Element concentrations in the extracts were measured by ICP-OES (Optima 4300 DV, Perkin Elmer Instruments, Norwalk, USA).

### Carbon stock assessment

Aboveground tree carbon was calculated using allometric equations^74^ and assuming a 50% carbon content in aboveground biomass^75, 76^. Dbh values were converted to those of diameter at 50 cm height using the equation:

6 $${\rm{Diameter}}\,{\rm{of}}\,{\rm{bole}}\,{\rm{at}}\,50\,{\rm{cm}}=1.1613\times DBH+0.4628$$

The equation used to predict bole diameter at 50 cm had reasonable accuracy (R^2^ = 0.77; Fig. S4). These values, as well as tree height values, were then submitted to the allometric equation of Vasquez *et al*.^74^:

7 $${\rm{Biomass}}=-16.51+40.26\times {\rm{tree}}\,{\rm{height}}\,(\mathrm{log}\,10)+9.30\times {\rm{tree}}\,{\rm{diameter}}\,(\mathrm{log}\,10)$$

SOC and root carbon were calculated from soil profiles in the four habitat types. Aboveground tree carbon, root carbon, and SOC were then averaged to give carbon stocks per m^2^ for the four habitat types (Table S8). To get current landscape carbon stocks, we extrapolated the min., max. lower and upper quartile and mean values per m^2^ of the carbon stocks over the area of the Cordillera Urubamba above 4200 m a.s.l. occupied by the four habitat types (Table S1). We did not investigate soil inorganic carbon, although this is not so relevant because CO_2_ is released when dissolved, but consumed again during precipitation. Aboveground herbaceous biomass was also not investigated, although this has a comparatively insignificant carbon stock^74, 76^. Potential landscape carbon stocks were calculated using the same approach but assuming that zonal vegetation of a landscape unaffected by humans would host the same proportion of forest-grassland that is found in inaccessible areas (Table S1).

### Data analysis

Mixed effects models were used to compare between inaccessible and accessible forest and grassland habitat types, including the fixed effect ‘human impact’, with the two levels ‘inaccessible’ and ‘accessible’. Analyses of 2 × 2 m^2^ plot data and soil analyses also included the fixed effects ‘vegetation type’, with the two levels ‘forest’ and ‘grassland’, and the interaction between ‘human impact’ and ‘vegetation type’. In analyses of 2 × 2 m^2^ plot data and soil analyses, the factor ‘site’, with eight levels corresponding to the eight sites studied, was treated as a random effect. Analyses of forest plot data included the random effects ‘elevation’, ‘tree species’ and ‘Cordillera’ (i.e. Urubamba or Vilcabamba). Analysis of lichen epiphyte species richness comparing standing deadwood and live *Polylepis* trees was done with the fixed effect ‘substrate type’, with two levels ‘deadwood’ and ‘live’, while the factor ‘site’, with four levels corresponding to the sites 1–4 of the Cordillera Urubamba, was treated as a random effect.

Generalized linear mixed effects model (GLMM) fitting and inference followed Bolker *et al*.^77^. Where data was normally distributed with homogeneity of variance, or could be transformed to achieve this, linear mixed effects models (LMM’s) were used, being fit using restricted maximum likelihood estimation (REML) t-tests using Satterthwaite approximations to degrees of freedom (Tables S12, S14 and S16–S18). GLMM’s with Laplace approximation were fit for data that could not be successfully transformed (Tables S13 and S15). In all LMM analyses, quantitatively similar results were obtained when running Gaussian GLMM with Laplace approximation as well as non-parametric Kruskal-Wallis tests and so only the LMM results are shown. GLMM’s were fit using R package ‘glmmadmb’ version 0.8.0 and LMM’s using R package ‘lmerTest’ version 1.0. Data was checked for normality using the Shapiro-Wilk normality test and non-normal data was transformed following Webster^78^. Proportional and percentage cover data were logit transformed. Homogeneity of variance was checked using the Bartlett test and, when non-normally distributed, the Levene and Fligner-Killeen tests were used.

To identify the vascular plant or epiphytic lichen species characteristic of each habitat type, whether inaccessible or accessible forest and grassland or standing deadwood or live trees, respectively, and to evaluate the proportion of these indicator species that were undescribed or introduced, Indicator Species Analyses were performed using the Indval method^79^ in R package ‘labdsv’ version 1.5–0.

Plant trait data was subjected to PCA from which 8 scaled principal components that represented 71.0% of the variation in the data were extracted for each species. Each principal component related to different morphologies and/or strategies to cope with grazing/burning (Table S6). These principal component values were then scored per species for each 2 × 2 m^2^ plot and mean values were calculated per plot. The mean values per plot of each principal component were then compared between the four habitat types using Gaussian GLMM’s with identity link function (Table S16). All analyses were performed in R 3.1.2 (R Foundation for Statistical Computing, Vienna, Austria).

## Electronic supplementary material

Supplementary Information
