# Supplementary material for: Relict high-Andean ecosystems challenge our concepts of naturalness and human impact
Source: Sci Rep. 2017 Jun 13;7:3334. doi: 10.1038/s41598-017-03500-7 (PMC5469861; doi:10.1038/s41598-017-03500-7)
Supplement: Supplementary file 1 — Supplementary Information [file 41598_2017_3500_MOESM1_ESM.pdf]

Supplementary information for

Relict high-Andean ecosystems challenge our concepts of naturalness and human impact

Steven P. Sylvester[1,2]*, Felix Heitkamp[3], Mitsy D.P.V. Sylvester[1,4], Hermann F. Jungkunst[5], Harrie J.M. Sipman[6], Johanna M. Toivonen[7], Carlos A. Gonzales Inca[8], Juan C. Ospina[9], Michael Kessler[1]

[1]Department of Systematic and Evolutionary Botany, University of Zurich, Switzerland.
[2]Department of Geography, Philipps-Universität Marburg, Germany.
[3]Physical Geography, Georg-August-Universität Göttingen, Germany.
[4]Universidad Nacional del San Antonio Abad del Cusco, Cusco, Peru.
[5]Geoecology & Physical Geography, Universität Koblenz-Landau, Germany.
[6]Botanischer Garten und Botanisches Museum Berlin-Dahlem, Freie Universität Berlin, Germany.
[7]Department of Biology, University of Turku, Finland.
[8]Department of Geography and Geology, University of Turku, Finland.
[9]Instituto de Botánica Darwinion (ANCEFN-CONICET), Buenos Aires, Argentina.
*Correspondence to: steven_sylvester@hotmail.com.

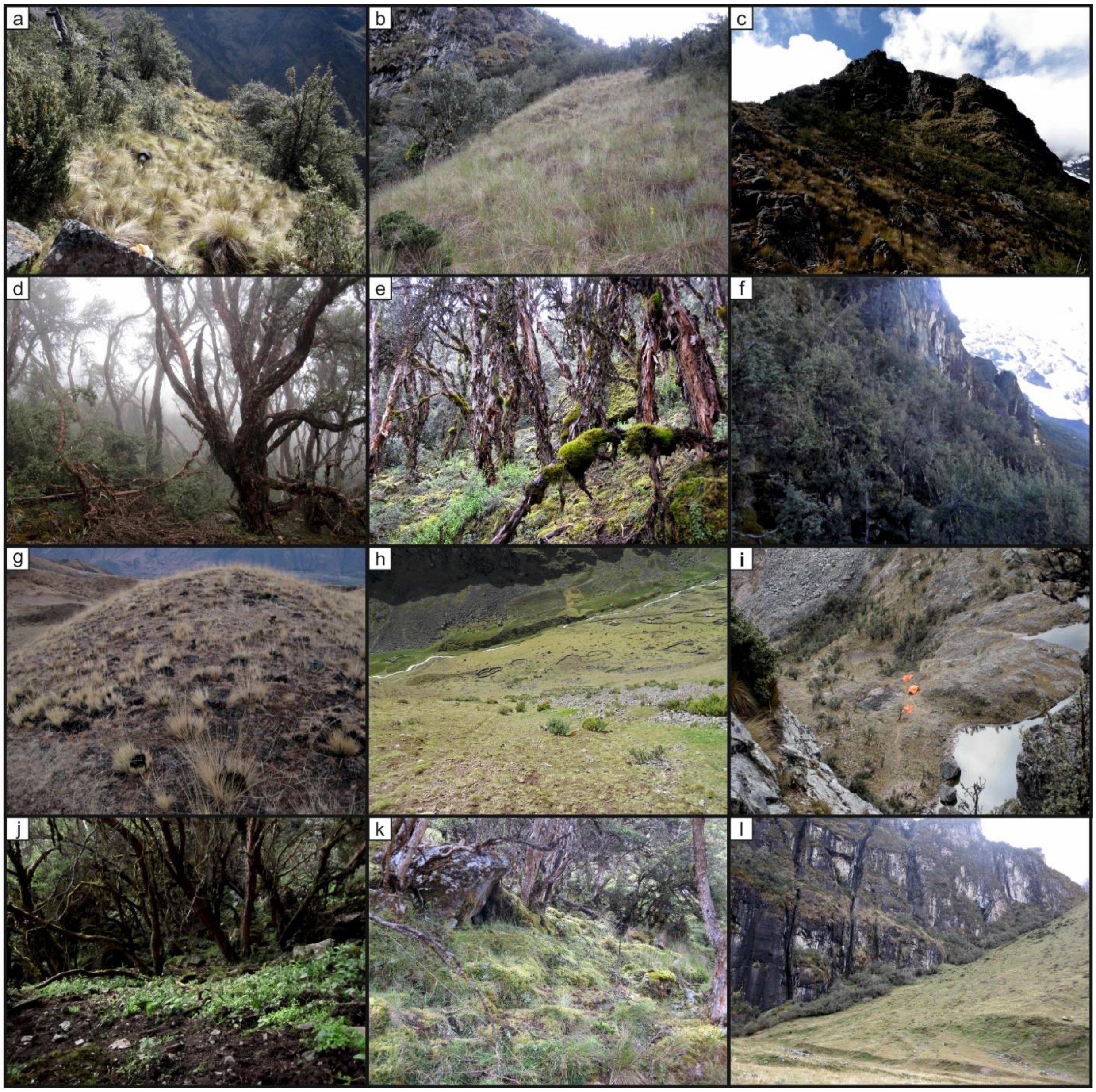

Fig. S1. Impression of the study sites showing examples of inaccessible (a-f) and accessible (g-l) vegetation. a, Inaccessible grassland of the Cordillera Urubamba dominated by undescribed tussock grasses, *Calamagrostis* sp. nov. 1 and *Festuca linigluma* J.C. Ospina, S.P. Sylvester and M.D.P.V. Sylvester[29]; **b,** Inaccessible grassland of the Cordillera Vilcabamba dominated by undescribed tussock grasses, *Calamagrostis* sp. nov. 1 and *Festuca proceroides* J.C. Ospina, S.P. Sylvester and M.D.P.V. Sylvester ined.; **c-f,** Inaccessible open forest and grassland (c), and closed forest (f) of the Cordillera Vilcabamba dominated by *Polylepis pepei* and *Gynoxys cuzcoensis* trees, and inaccessible forest stands of the Cordillera Urubamba dominated by *Polylepis subsericans* trees (d-e); **g-h,** Accessible grassland showing the dominance of short forbs as well as burnt tussocks; **i,** birds-eye view of an accessible site showing degraded open forest and grassland; **j-k,** Accessible forest showing either a high level of erosion from trampling by cattle (j) or a dominance of short grazing-adapted forbs (k); **l,** Example of an accessible forest remnant. Photographs taken by S.P. Sylvester.

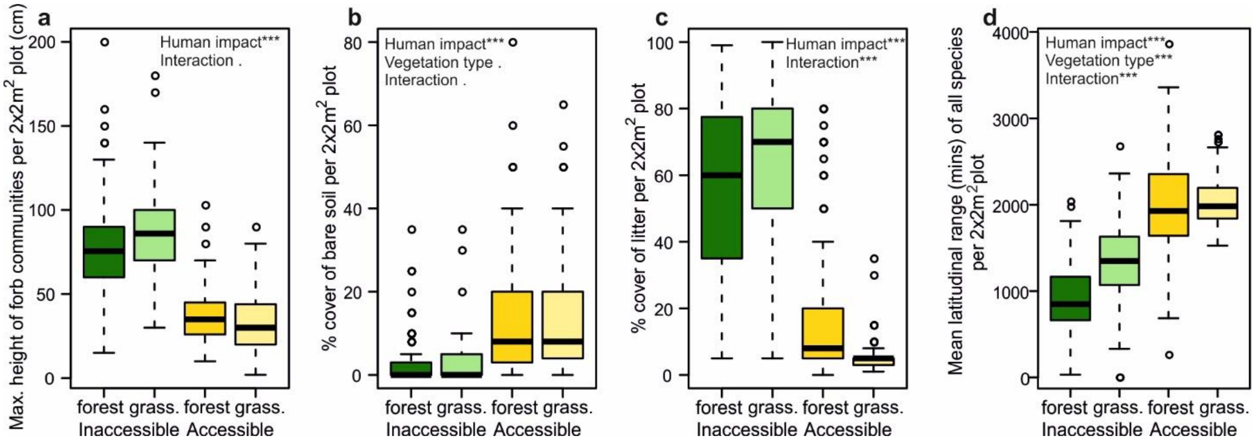

Fig. S2. Differences in vegetation structure (a-c) and latitudinal range-size of plant species per plot (d) between accessible and inaccessible forest and grassland habitat types. (a-d) Maximum height of herbaceous vegetation (a), cover of bare soil (b) and litter (c), and average latitudinal amplitude of all plant species (d) per $2x2m^2$ plot in the different habitat types. Significant relationships between the structural properties and human impact, found upon analysis using GLMM's, are noted within the plots (***=p<0.001, . =p<0.1; Tables S13). Sample size: Inaccessible forest 160, Inaccessible grassland 83, Accessible forest 118, Accessible grassland 111.

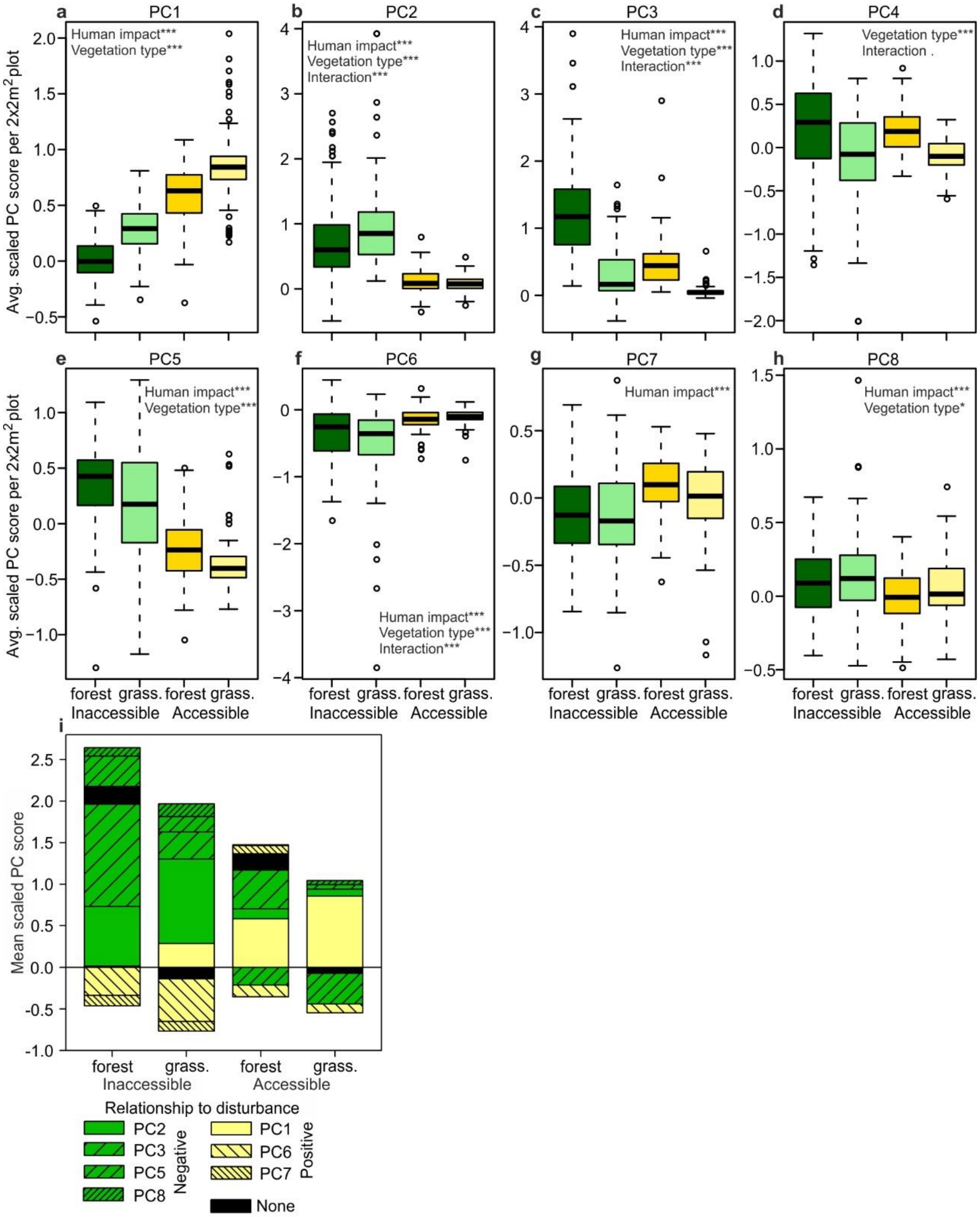

Fig. S3. Comparison of the scaled principal component (PC) scores, referring to plant trait syndromes (Table S6), averaged over all species per 2x2m^2 plot for the different habitat types. a-h, Differences in the mean scores of each individual PC per plot, between the habitat types. **i,** Comparison of the means of the 8 scaled principal components, with their relationship to disturbance shown by the colouring green (negative) and light yellow (positive). Significant relationships between the structural properties and human impact, found upon analysis using LMM's, are noted within plots a-h (***=p<0.001, *=p<0.05, . =p<0.1; Table S16). Sample size: Inaccessible forest 160, Inaccessible grassland 83, Accessible forest 118, Accessible grassland 111.

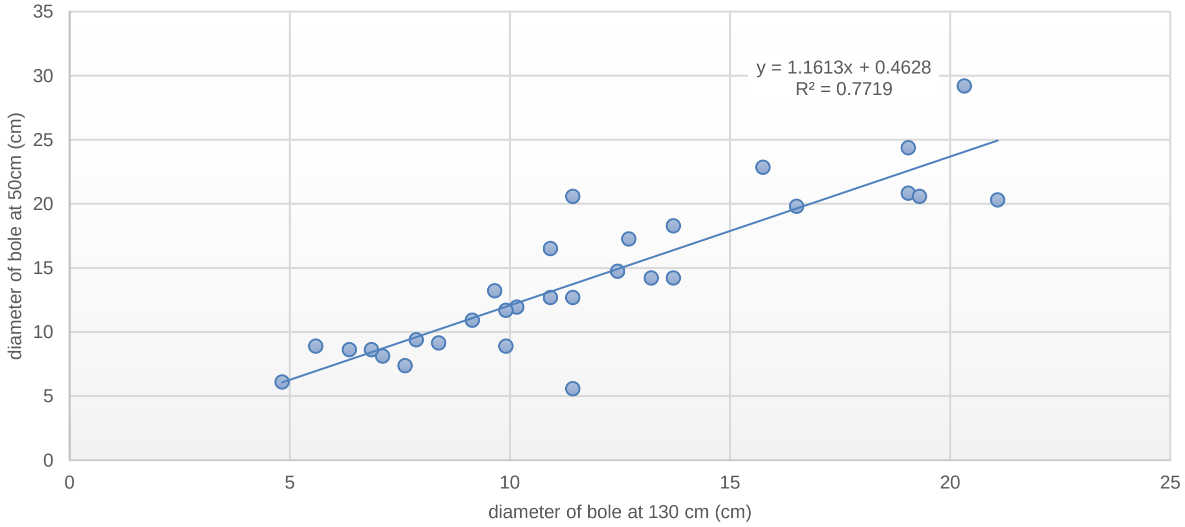

Fig. S4. Linear model for predicting tree bole diameter at 50cm using dbh values. To allow our data to be fitted to the allometric equation proposed by Vasquez et al.[74], who measure tree diameter at 50 cm from the ground, we created a linear model to extrapolate dbh data to that from 50 cm from the ground. To do this, 15 trees of *Polylepis racemosa* and 15 trees of *Polylepis incana* Kunth of different sizes were measured at 130 cm and 50 cm from ground level. These trees were growing in the vicinity of Cusco. Tree bole diameters at 50 and 130 cm were then plotted against each other and used to generate the linear model.

Table S1. Area and proportion of accessible and inaccessible areas of the Cordillera Urubamba covered by zonal grassland and forest or azonal habitat.

Human impact	Class	Area (m^2)	% cover	% zonal vegetation
Inaccessible	Grassland	419895	28.23	29.67
	Forest	995240	66.90	70.33
	Azonal	72496	4.87	
	Total	1487631		
Accessible	Grassland	69804605	53.22	92.49
	Forest	5669958	4.32	7.51
	Azonal	55697814	42.46	
	Total	1.31E+08		

Table S2. Undescribed species and habitat type where predominantly found with associated indicator species analysis[79] (indval) value and specimen information.

Family	New species to science	Habitat type where predominantly found	indval value	S.P. Sylvester specimen collection numbers made from study area (specimen duplicates are held in the CUZ, Z and LPB herbaria, amongst others)
Asteraceae	*Senecio* sp.nov.	Inaccessible forest	**0.13****	1458, 1604, 1865, 2111
Caprifoliaceae	*Valeriana vilcabambensis* S.P. Sylvester & Barrie ined.	Inaccessible forest	**0.10*****	240, 1418, 1465, 1519, 1866, 1872, 1879, 2110
Caryophyllaceae	*Stellaria* sp.nov.	Inaccessible forest	0.01	682, 1688
Orobanchaceae	*Bartsia lydiae* S.P. Sylvester	Inaccessible forest	0.02	127, 464, 680, 815, 878, 939, 1017, 1026, 1407, 1649, 1730, 1754
Poaceae	*Calamagrostis* sp.nov. 1	Inaccessible forest	**0.18*****	664, 692, 803, 1015, 1048, 1289, 1290
Poaceae	*Festuca linigluma* J.C. Ospina, S.P. Sylvester & M.D.P.V. Sylvester	Inaccessible forest	**0.17****	745, 778, 802, 871, 890, 1014, 1047, 1100, 1264, 1291, 1325, 1633, 1644, 2244
Poaceae	*Festuca proceroides* J.C. Ospina, S.P. Sylvester & M.D.P.V. Sylvester ined.	Inaccessible forest	**0.15*****	233, 257, 295, 330, 353, 1420, 1421, 1455, 1506, 1507, 1583, 1848, 1898, 1899, 1902, 1903, 1908, 2120, 2139, 2200
Poaceae	*Poa urubambensis* S.P. Sylvester & Soreng	Inaccessible forest	**0.07****	812, 869, 1317, 1636, 1637, 1695, 403, 1727
Poaceae	*Calamagrostis* sp.nov. 2	Inaccessible grassland	0.01	1053, 1067, 1906
Poaceae	*Festuca calcana* J.C. Ospina, S.P. Sylvester & M.D.P.V. Sylvester ined.	Inaccessible grassland	0.01	387, 1204, 1680
Polypodiaceae	*Moranopteris inaccessa* Sundue & S.P. Sylvester	Inaccessible grassland	0.01	300, 1628a
Asteraceae	*Werneria* sp.nov.	Accessible forest	**0.04***	197, 603, 617, 1055, 1078
Campanulaceae	*Lysipomia mitsyae* S.P. Sylvester & D. Quandt	Accessible grassland	**0.27*****	823, 1417
Gentianaceae	*Gentianella viridiflora* S. Pfanzelt & S.P. Sylvester	Accessible grassland	**0.16*****	1951
Iridaceae	*Cardenanthus* sp.nov.	Accessible grassland	**0.33*****	855, 1383, 1416

Inaccessible forest, where the majority of undescribed species were found, is highlighted in grey. Species with significant ($p < 0.05$) indval values are highlighted in bold. Signif. codes: '***'=p<0.001, '**'=p<0.01,'*'=p<0.05. . Species considered important components of each habitat type were given larger indval values and those seen to be more restricted to a certain habitat type were given higher significance values for that habitat type.

Table S3. Comparison of indicator plant species and the proportion which are undescribed or introduced

Habitat type	Total No. of indicator species	Proportion of undescribed species as indicators (%)	Proportion of introduced species as indicators (%)
Inaccessible Forest	24	25	0
Inaccessible Grassland	20	0	0
Accessible Forest	57	3.5	10.5
Accessible Grassland	82	2.4	0

Comparison between the four habitat types of the number of significant ($p < 0.05$) vascular plant indicator species following indval analysis, and the proportion of this number which are undescribed or introduced. Detailed results are found in Table S4.

Table S4. Indicator species analysis[79] (indval) values of plants comparing inaccessible and accessible forest and grassland habitat.

Inaccessible forest indicator species	indval	Inaccessible grassland indicator species	indval
***Polylepis subsericans* J.F.Macbr.**	0.31***	***Calamagrostis tarmensis* Pilg.**	0.30***
***Ourisia chamaedrifolia* Benth.**	0.21***	***Perezia pungens* (Bonpl.) Less.**	0.14***
***Luzula gigantea* Desv.**	0.21***	***Valeriana micropterina* Wedd.**	0.13***
***Gynoxys nitida* Muschl.**	0.19***	***Bartsia thiantha* Diels**	0.12***
***Calamagrostis* sp.nov. 1**	0.18***	***Baccharis johnwurdackiana* H. Rob.**	0.12***
***Pentacalia dictyophlebia* (Greenm.) Cuatrec.**	0.18***	***Pernettya prostrata* (Cav.) DC.**	0.11***
***Ribes brachybotrys* (Wedd.) Jancz.**	0.16***	***Baccharis tola* Phil.**	0.11***
***Festuca proceroides* J.C. Ospina & S.P. Sylvester sp.nov. ined.**	0.15***	***Achyrocline alata* (Kunth) DC.**	0.09***
***Calamagrostis intermedia* (J. Presl) Steud.**	0.13***	***Calamagrostis recta* (Kunth) Trin. ex Steud.**	0.09***
***Cerastium subspicatum* Wedd.**	0.12***	***Austrolycopodium magellanicum* (P. Beauv.) Holub**	0.06**
***Valeriana vilcabambensis* S.P. Sylvester & Barrie sp.nov. ined.**	0.10***	***Carex pichinchensis* Kunth**	0.10*
***Bomarea dulcis* (Hook.) Beauverd**	0.23*	***Calamagrostis rigida* (Kunth) Trin. ex Steud.**	0.09*
***Festuca linigluma* J.C. Ospina, S.P. Sylvester & M.D.P.V. Sylvester sp.nov.**	0.17*	***Valeriana mandoniana (Wedd.) Höck***	0.07*
***Senecio praeruptorum* Sch. Bip. ex Klatt**	0.13*	***Niphogeton dissecta* (Benth.) J.F. Macbr.**	0.06*
***Senecio* sp.nov.**	0.13*	***Ranunculus krapfia* DC. Ex Deless**	0.05*
***Gynoxys cuzcoensis* Cuatrec.**	0.11*	***Misbrookea strigosissima* (A. Gray) V.A. Funk**	0.04*
***Chersodoma antennaria* (Wedd.) Cabrera**	0.10*	***Hieracium streptochaetum* Zahn**	0.03*
***Senecio rhizomatus* Rusby**	0.08*	***Senecio melanocalyx* Cuatrec.**	0.02*
***Melpomene peruviana* (Desv.) A.R. Sm. & R.C. Moran**	0.07*	***Lysipomia glandulifera* (Schltdl. ex Wedd.) Schltdl. ex E. Wimm.**	0.02*
***Oxalis phaeotricha* Diels**	0.07*	***Calceolaria virgata* Ruiz & Pav.**	0.02*
***Poa urubambensis* S.P.Sylvester & Soreng sp.nov.**	0.07*	*Berberidaceae* sp.1	0.02.
***Senecio hohenackeri* Sch. Bip.**	0.07*	*Polystichum cochleatum* (Klotzsch) Hieron.	0.02.
***Gentianella scarlatiflora* (Gilg) J.S.Pringle**	0.04*	*Werneria villosa* A. Gray	0.05
***Tristerix longebracteatus* (Desr.) Barlow & Wiens**	0.04*	*Anatherostipa hans-meyeri* (Pilg.) Peñail.	0.04
Poa cf.huancavelicae Tovar	0.06.	*Werneria orbignyana* Wedd.	0.04
Agrostis perennans (Walter) Tuck.	0.05.	*Senecio chrysolepis* Phil.	0.04
Bartsia flava Molau	0.04.	*Ageratina glechonophylla* (Less.) R.M. King. H. Rob.	0.03
Salpichroa glandulosa (Hook.) Miers	0.04	*Belonanthus hispidus* (Wedd.) Graebn.	0.03
Fuchsia apetala Ruiz & Pav.	0.03	*Halenia phyteumoides* Gilg (syn.= *Halenia weberbaueri* C.K. Allen)	0.02
Melpomene personata Lehnert	0.02	*Achyrocline ramosissima* Britton	0.02
Bartsia lydiae S.P. Sylvester sp.nov.	0.02	*Senecio genisianus* Cuatrec.	0.02
Senecio hastatifolius Cabrera	0.02	*Poa cf.trollii* (Pilg.) Refulio (syn. = *Dissanthelium trollii* Pilg.)	0.01
Baccharis caespitosa (Ruiz &Pav.) Pers.	0.02	*Werneria plantaginifolia* Wedd. ex Klatt	0.01
Festuca sp.1	0.01	*Gentianella thyrsoidea* (Hook.) Fabris	0.01
Bartsia elongata Wedd.	0.01	*Senecio flaccidifolius* Wedd.	0.01
Belloa schultzii (Wedd.) Cabrera	0.01	*Saxifraga magellanica* Poir.	0.01
Senecio adenophyllus Meyen. Walp.	0.01	*Caiophora contorta* (Desr.) C. Presl	0.01
Jamesonia imbricata (Sw.) Hook. & Grev.	0.01	*Senecio nutans* Sch.Bip.	0.01

Species with significant ($p < 0.05$) indval values are in bold. Undescribed species are highlighted in yellow and introduced species are highlighted in blue. Signif. codes: '***'=p<0.001, '**'=p<0.01,'*'=p<0.05, '.'=p<0.1. Species considered important components of each habitat type were given larger indval values and those seen to be more restricted to a certain habitat type were given higher significance values for that habitat type.

Table S4 cont. Indicator species analysis[79] (indval) values of plants comparing inaccessible and accessible forest and grassland habitat.

Inaccessible forest indicator species	indval	Inaccessible grassland indicator species	indval
Stellaria sp.nov.	0.01	*Werneria staticifolia* Sch.Bip.	0.01
Erigeron rosulatus Wedd.	0.01	*Calamagrostis* sp.nov. 2	0.01
Berberis saxicola Lechler	0.01	*Miconia chionophila* Naudin	0.01
Calamagrostis cf.polygama (Griseb.) Parodi	0.01	*Campyloneurum asplundii* (C. Chr.) Ching	0.01
Lasiocephalus lingulatus Schltdl.	0.01	*Moranopteris inaccessa* Sundue & S.P. Sylvester sp.nov.	0.01
Loricaria thuyoides (Lam.) Sch.Bip.	0.01	*Valeriana radicata* Graebn.	0.01
Sisyrinchium palmifolium L.	0.01	*Chaptalia rotundifolia* D. Don	0.01
Silene andicola Gillies ex Hook. & Arn.	0	*Olsynium junceum* (E. Mey. ex C. Presl) Goldblatt	0.01
		Festuca calcana J.C. Ospina & S.P. Sylvester sp.nov. ined.	0.01
**Accessible forest indicator species**	**indval**	**Accessible grassland indicator species**	**indval**
***Poa horridula* Pilg.**	**0.42*****	***Lachemilla pinnata* (Ruiz & Pav.) Rothm.**	**0.50*****
***Polylepis racemosa* Ruiz & Pav.**	**0.31*****	***Geranium sessiliflorum* Cav.**	**0.47*****
***Oreomyrrhis andicola* (Kunth) Endl. ex Hook. f.**	**0.29*****	***Hypochaeris meyeniana* (Walp.) Benth. & Hook. f. ex Griseb.**	**0.46*****
***Azorella multifida* (Ruiz & Pav.) Pers.**	**0.26*****	***Calamagrostis vicunarum* (Wedd.) Pilg.**	**0.44*****
***Polylepis pepei* B.B. Simpson**	**0.24*****	***Luzula racemosa* Desv.**	**0.44*****
***Gunnera magellanica* Lam.**	**0.23*****	***Gamochaeta americana* (Mill.) Wedd.**	**0.39*****
***Urtica magellanica* Juss. ex Poir.**	**0.23*****	***Sisyrinchium brevipes* Baker**	**0.37*****
***Poa annua* L.**	**0.22*****	***Oxalis oreocharis* Diels**	**0.37*****
***Gentianella rima* (D.Don ex G.Don) Fabris**	**0.21*****	***Agrostis breviculmis* Hitchc.**	**0.36*****
***Uncinia macrolepis* Decne.**	**0.21*****	***Calamagrostis heterophylla* (Wedd.) Pilg.**	**0.35*****
***Cotula mexicana* (DC.) Cabrera**	**0.20*****	***Oenothera multicaulis* Ruiz & Pav.**	**0.33*****
***Ranunculus repens* L.**	**0.19*****	***Cardenanthus* sp.nov.**	**0.33*****
***Werneria nubigena* Kunth**	**0.19*****	***Aciachne acicularis* Lægaard**	**0.30*****
***Geranium core core* Steud.**	**0.18*****	***Lysipomia mitsyae* S.P. Sylvester & D. Quandt sp.nov.**	**0.27*****
***Poa gymnantha* Pilg.**	**0.18*****	***Galium corymbosum* Ruiz & Pav.**	**0.27*****
***Stellaria media* (L.) Vill.**	**0.16*****	***Belloa piptolepis* (Wedd.) Cabrera**	**0.27*****
***Stellaria weddellii* Pedersen**	**0.16*****	***Carex ecuadorica* K.k.**	**0.26*****
***Lachemilla andina* (L.M. Perry) Rothm.**	**0.16*****	***Azorella biloba* (Schltdl.) Wedd.**	**0.24*****
***Arenaria soratensis* Rohrb.**	**0.16*****	***Crassula closiana* (Gay) Reiche**	**0.23*****
***Baccharis alpina* Kunth**	**0.15*****	***Peperomia verruculosa* Dahlst. ex A.W. Hill**	**0.22*****
***Veronica arvensis* L.**	**0.15*****	***Novenia acaulis* (Benth. & Hook. f. ex B.D. Jacks.) S.E. Freire & F.H. Hellw.**	**0.22*****
***Sisyrinchium jamesonii* Baker**	**0.13*****	***Muhlenbergia peruviana* (P. Beauv.) Steud.**	**0.21*****
***Halenia umbellata* (Ruiz & Pav.) Gilg**	**0.13*****	***Cerastium crassipes* Bartl.**	**0.17*****
***Stellaria cuspidata* Willd. ex Schltdl.**	**0.12*****	***Lupinus microphyllus* Desr.**	**0.17*****
***Bromus lanatus* Kunth**	**0.11*****	***Paspalum pygmaeum* Hack.**	**0.17*****
***Senecio peruensis* Cuatrec.**	**0.11*****	***Gentianella peruviana* (Griseb.) Fabris**	**0.16*****
***Lysipomia laciniata* A. DC.**	**0.11*****	***Gentianella* sp.1**	**0.16*****
***Gentianella viridiflora* S. Pfanzelt & S.P. Sylvester sp.nov.**	**0.08*****	***Festuca casapaltensis* J.Ball**	**0.15*****

Species with significant ($p < 0.05$) indval values are in bold. Undescribed species are highlighted in yellow and introduced species are highlighted in blue. Signif. codes: '***'=p<0.001, '**'=p<0.01,'*'=p<0.05, '.'=p<0.1. Species considered important components of each habitat type were given larger indval values and those seen to be more restricted to a certain habitat type were given higher significance values for that habitat type.

Table S4 cont. Indicator species analysis[79] (indval) values of plants comparing inaccessible and accessible forest and grassland habitat.

Accessible forest indicator species	indval	Accessible grassland indicator species	indval
***Festuca tenuiculmis* Tovar**	**0.06*****	***Muehlenbeckia volcanica* (Benth.) Endl.**	**0.15*****
***Solanum tuberosum* L.**	**0.06*****	***Myrosmodes paludosum* (Rchb. f.) P. Ortiz**	**0.14*****
***Poa subspicata* (J. Presl) Kunth**	**0.13***	***Cyperus seslerioides* Kunth**	**0.14*****
***Calamagrostis deserticola* (Phil.) Phil.**	**0.09***	***Calamagrostis rigescens* (J. Presl) Scribn.**	**0.13*****
***Plantago australis* Lam.**	**0.07***	***Arenaria digyna* Willd. ex Schltdl.**	**0.12*****
***Belloa longifolia* (Cuatrec.) Sagast. & Dillon**	**0.07***	***Poa serpaiana* Refulio**	**0.11*****
***Agrostis tolucensis* Kunth**	**0.07***	***Ophioglossum crotalophoroides* Walter**	**0.10*****
***Perezia ciliosa* (Phil.) Reiche**	**0.06***	***Plantago lamprophylla* Pilg.**	**0.10*****
***Carex bonplandii* Kunth**	**0.06***	***Lachemilla aphanoides* (Mutis ex L. f.) Rothm.**	**0.09*****
***Erigeron cf.chionophilus* Wedd.**	**0.06***	***Oxalis nubigena* Walp.**	**0.08*****
***Astragalus uniflorus* DC.**	**0.06***	***Lupinus herzogii* Ulbr.**	**0.08*****
***Werneria pectinata* Lingelsh.**	**0.05***	***Aphanactis villosa* S. F. Blake**	**0.08*****
***Polystichum nudicaule* Rosenst.**	**0.05***	***Poa macusaniensis* (E.H.L. Krause) Refulio**	**0.08*****
***Cystopteris fragilis* (L.) Bernh.**	**0.05***	***Geranium sibbaldioides* Benth.**	**0.08*****
***Bromus modestus* Renvoize**	**0.05***	***Lachemilla fulvescens* (L.M. Perry) Rothm.**	**0.07*****
***Senecio modestus* Wedd.**	**0.05***	***Wahlenbergia peruviana* A.Gray**	**0.07*****
***Halenia caespitosa* Gilg**	**0.05***	***Gentiana sedifolia* Kunth**	**0.07*****
***Cerastium glomeratum* Thuill.**	**0.05***	***Gnaphalium melanosphaeroides* Sch. Bip. ex Wedd.**	**0.07*****
***Festuca soukupii* Stancik**	**0.04***	***Trifolium amabile* Kunth**	**0.07*****
***Plagiobothrys humilis* (Ruiz & Pav.) I.M. Johnst.**	**0.04***	***Solanum acaule* Bitter**	**0.07*****
***Cardamine bonariensis* Pers.**	**0.04***	***Acaena cylindristachya* Ruiz & Pav.**	**0.05*****
***Valeriana pycnantha* A. Gray**	**0.04***	***Oreobolopsis tepalifera* T. Koyama & Guagl.**	**0.18****
***Werneria caespitosa* Wedd.**	**0.04***	***Perezia pinnatifida* (Bonpl.) Wedd.**	**0.10***
***Werneria* sp.nov.**	**0.04***	***Werneria pygmaea* Gillies ex Hook. & Arn.**	**0.08***
***Lachemilla orbiculata* (Ruiz & Pav.) Rydb.**	**0.04***	***Gentianella cf.persquarrosa* (Reimers) J.S. Pringle**	**0.08***
***Capsella bursa pastoris* (L.) Medik.**	**0.03***	***Poa perligulata* Pilg.**	**0.07***
***Hydrocotyle filipes* Mathias**	**0.03***	***Festuca cf.rigescens* (J. Presl) Kunth**	**0.07***
***Asplenium castaneum* Schltdl. & Cham.**	**0.03***	***Bartsia peruviana* Walp.**	**0.07***
***Bromus catharticus* Vahl**	**0.03***	***Calandrinia acaulis* Kunth**	**0.07***
Oritrophium hieracioides (Wedd.) Cuatrec.	0.06.	***Tarasa urbaniana* (Ulbr.) Krapov.**	**0.06***
Lachemilla tanacetifolia Rothm.	0.04.	***Hordeum muticum* J. Presl**	**0.06***
Montia fontana L.	0.03.	***Hypochaeris taraxacoides* Ball**	**0.06***
Plantago rigida Kunth	0.03.	***Aphanes parvula* Gutte**	**0.06***
Poa swallenii Refulio	0.03.	***Muhlenbergia ligularis* (Hack.) Hitchc.**	**0.06***
Draba cuzcoensis O.E.Schulz	0.03.	***Lupinus pulvinaris* Ulbr.**	**0.06***
Veronica opaca Fr.	0.02.	***Belloa kunthiana* (DC.) Anderb. & S.E. Freire**	**0.06***
Nototriche longirostris (Wedd.) A.W. Hill	0.02.	***Berberis lutea* Ruiz & Pav.**	**0.05***
Castilleja virgatoides Edwin	0.04	***Castilleja pumila* (Benth.) Wedd.**	**0.05***

Species with significant ($p < 0.05$) indval values are in bold. Undescribed species are highlighted in yellow and introduced species are highlighted in blue. Signif. codes: '***'=p<0.001, '**'=p<0.01,'*'=p<0.05, '.'=p<0.1. Species considered important components of each habitat type were given larger indval values and those seen to be more restricted to a certain habitat type were given higher significance values for that habitat type.

Table S4 cont. Indicator species analysis[79] (indval) values of plants comparing inaccessible and accessible forest and grassland habitat.

Accessible forest indicator species	indval	Accessible grassland indicator species	indval
Baccharis papillosa Rusby	0.03	***Senecio candollei*** **Wedd.**	**0.05***
Festuca dolichophylla J.Presl	0.03	***Nassella mexicana*** **(Hitchc.) R.W. Pohl**	**0.05***
Nassella inconspicua (J. Presl) Barkworth	0.02	***Perezia multiflora*** **(Bonpl.) Less.**	**0.04***
Noticastrum marginatum (H.B.K.) Cuatr.	0.02	***Facelis plumosa*** **(Wedd.) Sch.Bip.**	**0.04***
Werneria aretioides Wedd.	0.02	***Paronychia muschleri*** **Chaudhri**	**0.04***
Vulpia myuros (L.) C.C. Gmel.	0.02	***Bartsia pauciflora*** **Molau**	**0.03***
Calceolaria engleriana Kraenzl.	0.02	***Carex boliviensis*** **Van Heurck & M.ll. Arg.**	**0.03***
Polypodium chrysolepis Hook.	0.02	***Calamagrostis amoena var. festucoides*** **(Wedd.) Soreng**	**0.03***
Brayopsis alpaminae Gilg & Muschl. subsp. alpaminae	0.02	***Bidens andicola*** **Kunth**	**0.03***
Thelypteris	0.02	***Calceolaria scapiflora*** **(Ruiz & Pav.) Benth.**	**0.03***
Trisetum spicatum (L.) K. Richt.	0.02	***Hypericum brevistylum*** **Choisy**	**0.03***
Epilobium denticulatum Ruiz & Pav.	0.02	***Luciliocline subspicata*** **(Wedd.) Anderb. & S.E.Freire**	**0.03***
Mimulus glabratus Kunth	0.02	***Urtica flabellata*** **Kunth**	**0.03***
Valeriana mandonii Britton	0.02	***Poa calycina*** **(J. Presl) Kunth**	**0.03***
Poa infirma Kunth	0.02	***Trichophorum rigidum*** **(Boeckeler) Goetgh. Muasya & D.A. Simpson**	**0.03***
Cerastium mollissimum Poir.	0.01	***Crassula connata*** **(Ruiz & Pav.) A.Berger**	**0.03***
Draba werffii Al Shehbaz	0.01	*Gnaphalium badium* Wedd.	0.04.
Bowlesia flabilis J.F. Macbr.	0.01	*Lepidium abrotanifolium* Turcz.	0.03.
Senecio canescens (Humb. & Bonpl.) Cuatrec.	0.01	*Lupinus soratensis* Rusby	0.02.
Plantago tubulosa Decne.	0.01	*Hedeoma mandoniana* Wedd.	0.02.
Polystichum orbiculatum (Desv.) J. Rémy & Fée	0.01	*Gamochaeta purpurea* (L.) Cabrera	0.02.
Oritrophium sp.	0.01	*Festuca peruviana* Infantes	0.02.
Aciachne pulvinata Benth.	0.01	*Festuca asplundii* E.B. Alexeev	0.02.
Urtica urens L.	0.01	*Distichia muscoides* Nees. Meyen.	0.02.
Hypochaeris sessiliflora Kunth	0.01	*Castilleja nubigena* Kunth	0.02.
Calamagrostis ovata (J. Presl) Steud.	0.01	*Carex jamesonii* Boott	0.02.
Ribes bolivianum Jancz.	0.01	*Alstroemeria pygmaea* Herb.	0.02.
Clinopodium brevicalyx (Epling) Harley & A.Granda	0.01	*Agrostis trichodes* (Kunth) Roem. & Schult.	0.02.
Oxalis conorrhiza Jacq.	0.01	*Acaena elongata* L.	0.02.
Colobanthus quitensis (Kunth) Bartl.	0.01	*Paranephelius ovatus* A.Gray ex Wedd.	0.02
Senecio sp.1	0.01	*Lupinus chlorolepis* C.P. Sm.	0.02
Phyllactis pulvinata Rauh. Willer	0.01	*Oritrophium limnophilum* (Sch.Bip.) Cuatrec.	0.02
Asplenium haenkeanum (C. Presl) Hieron.	0.01	*Bartsia pyricarpa* Molau	0.02
Asplenium peruvianum Desv.	0.01	*Arcytophyllum filiforme* (Ruiz & Pav.) Standl.	0.02
Festuca humilior Nees. Meyen	0.01	*Rumex acetosella* L.	0.01
Elaphoglossum mathewsii (Fée) T. Moore	0.01	*Veronica serpyllifolia* L.	0.01
Melpomene moniliformis (Lagasca ex. Sw.) A.R.Sm. & R.C.Moran	0.01	*Bartsia stricta* (Kunth) Benth.	0.01
Elaphoglossum hartwegii (Fée) T. Moore	0.01	*Microsteris gracilis* (Douglas ex Hook.) Greene	0.01

Species with significant (p <0.05) indval values are in bold. Undescribed species are highlighted in yellow and introduced species are highlighted in blue. Signif. codes: '***'=p<0.001, '**'=p<0.01,'*'=p<0.05, '.'=p<0.1. Species considered important components of each habitat type were given larger indval values and those seen to be more restricted to a certain habitat type were given higher significance values for that habitat type.

Table S4 cont. Indicator species analysis[79] (indval) values of plants comparing inaccessible and accessible forest and grassland habitat.

Accessible forest indicator species	indval	Accessible grassland indicator species	indval
Cardamine vulgaris Phil.	0.01	*Eleocharis albibracteata* Nees & Meyen ex Kunth	0.01
Descurainia myriophylla (Willd. ex DC.) R.E. Fr. (Synonym= D.mabridei O.E. Schulz)	0.01	*Woodsia montevidensis* (Spreng.) Hieron.	0.01
Calamagrostis orbignyana (Wedd.) Wedd. ex Pilg.	0	*Gamochaeta longipedicellata* Cabrera	0.01
Uncinia phleoides (Cav.) Pers.	0	*Viola pygmaea* Juss. ex Poir	0.01
		Phlegmariurus crassus (Humb. & Bonpl. ex Willd.) B. Øllg.	0.01
		Lachemilla vulcanica (Schltdl. & Cham.) Rydb.	0.01
		Taraxacum officinale F.H. Wigg.	0.01
		Asteraceae sp.3	0.01
		Plagiobothrys congestus (Wedd.) I.M. Johnst.	0.01
		Potentilla dombeyi Nestl.	0.01
		Asplenium stoloniferum Bory	0.01
		Calamagrostis spicigera (J. Presl) Steud.	0.01
		Festuca inarticulata Oilg.	0.01
		Calamagrostis sp.3	0.01
		Cicendia quadrangularis (Dombey ex Lam.) Griseb.	0.01
		Soliva sessilis Ruiz & Pav.	0.01
		Calamagrostis fiebrigii Pilg.	0.01
		Peperomia sp.2	0.01
		Peperomia sp.1	0.01
		Descurainia athrocarpa (A. Gray) O.E. Schulz	0.01
		Lysipomia sphagnophila Griseb. ex Wedd.	0.01
		Senecio evacoides Sch.Bip. ex Wedd.	0.01
		Calamagrostis sclerantha Hack.	0.01
		Cotula australis (Sieber ex Spreng.) Hook. f.	0.01
		Agrostis meyenii Trin.	0.01
		Lupinus ballianus C.P. Sm.	0.01
		Ribes cuneifolium Ruiz & Pav.	0.01
		Brayopsis calycina (Desv.) Gilg & Muschl.	0
		Ephedra rupestris Benth.	0

Species with significant ($p < 0.05$) indval values are in bold. Undescribed species are highlighted in yellow and introduced species are highlighted in blue. Signif. codes: '***'=p<0.001, '**'=p<0.01,'*'=p<0.05, '.'=p<0.1. Species considered important components of each habitat type were given larger indval values and those seen to be more restricted to a certain habitat type were given higher significance values for that habitat type.

Table S5. Indicator species analysis[79] (indval) values of epiphytic lichen species comparing deadwood and live tree habitat.

Deadwood indicator species	indval	Live tree indicator species	indval
***Parmelinopsis subfatiscens* (Kurok.) Elix & Hale**	**0.29*** **	*Cetrariastrum* sp.	0.03.
***Normandina pulchella* (Borrer) Nyl.**	**0.26*** **	*Hypotrachyna bogotensis* (Vain.) Hale	0.02.
***Ramalina reducta* Krog & Swinscow**	**0.23*** **	*Usnea* sp.1	0.06
***Heterodermia parva* Morbeg**	**0.22*** **	*Cladonia borbonica* Nyl.	0.04
***Usnea fulvoreagens* (Räsänen) Räsänen**	**0.2*** **	*Chaenotheca brunneola* (Ach.) Müll. Arg.	0.04
***Caloplaca* sp.1**	**0.2*** **	*Chaenotheca furfuracea* (L.) Tibell	0.04
***Leptogium resupinans* Nyl.**	**0.2*** **	*Buellia rhizocarpica* Etayo, Giralt & Elix.	0.03
***Rinodina* sp.**	**0.18*** **	*Parmeliella incrassata* P.M. Jørg.	0.03
***Bacidia* sp.**	**0.14*** **	*Chaenotheca* aff. *chrysocephala* (Turner ex Ach.) Th. Fr.	0.02
***Heterodermia magellanica* (Zahlbr.) Winsc. & Krog**	**0.09*** **	*Cladonia pocillum* (Ach.) O.J. Rich.	0.02
***Chaenotheca chrysocephala* (Turner ex Ach.) Th. Fr.**	**0.21** **	*Sticta laevis* (Nyl.) Vain.	0.02
***Buellia* sp.1**	**0.17** **	*Sticta albocyphellata* Moncada & Lücking ined.	0.02
***Oropogon bicolor* Essl.**	**0.14** **	*Cladonia chlorophaea* (Flörke ex Sommerf.) Spreng.	0.02
***Cetrariastrum dubitans* Sipman**	**0.12** **	*Sticta paralimbata* Moncada & Lücking ined.	0.02
***Pannaria rubiginosa* (Thunb. ex Ach.) Delise**	**0.09** **	*Botryolepraria neotropica* Kukwa & Pérez-Ortega	0.02
***Amandinea* sp.1**	**0.08** **	*Cladonia subradiata* (Vain.) Sandst.	0.01
**Sp.1**	**0.07** **	*Sticta dioica* Moncada & Lücking ined.	0.01
***Cetrariastrum equadoriense* (R.Sant.) Sipman**	**0.13* **	*Chaenotheca* cf. *chlorella* (Ach.)Müll.Arg.	0.01
***Lecidea* sp.1**	**0.1* **	*Coenogonium* aff. *luteolum* (Kalb) Kalb & Lücking	0.01
***Leptogium andinum* P.M. Jørg.**	**0.09* **	*Dictyonema glabratum* (Spreng.) D. Hawksw.	0.01
***Oropogon loxensis* (Fée) Zukal**	**0.09* **	*Gyalideopsis* sp.	0.01
***Melanohalea subolivacea* (Nyl. ex Hasse) O. Blanco, A. Crespo, Divakar, Essl., D. Hawksw. & Lumbsch**	**0.08* **	*Hypogymnia* sp.	0.01
***Buellia* sp.2**	**0.08* **	*Hypotrachyna longiloba* (H. Magn.) C.W.Sm.	0.01
***Oropogon* sp.1**	**0.07* **	*Pertusaria subambigens* Dibben	0.01
***Collema glaucophthalmum* Nyl.**	**0.06* **	*Phyllopsora* sp.	0.01
***Scoliciosporum* sp.**	**0.05* **	Sp.7	0.01
***Buellia* sp.4**	**0.04* **	*Hypotrachyna* sp.	0.01
***Ochrolechia africana* Vain.**	**0.03* **	*Peltigera fibrilloides* (Gyeln.) Vitik.	0.01
Arthonia sp.1	0.09.	*Hypotrachyna meyeri* (Zahlbr.) Streimann	0.01
Leptogium punctulatum Nyl.	0.06.	*Chrysothrix* sp.	0.01
Opegrapha sp.	0.05.	*Parmelina cleefii* Sipman	0.01
Biatora sp.	0.04.	*Sticta tomentosa* (Sw.) Ach.	0.01
Agonimia tristicula (Nyl.) Zahlbr.	0.03.	*Sticta atroandensis* Moncada & Lücking	0.01
Cetrariastrum andense (Kärnefelt) Sipman	0.09	*Dimerella pineti* (Ach.) Vězda	0.01
Usnea wasmuthii Räsänen	0.08	*Sticta brevior* Moncada & Lücking	0.01
Leptogium cochleatum (Dicks.) P.M. Jørg. & P. James	0.08	*Sticta marilandia* Moncada & Lücking ined.	0.00
Sticta sp.5	0.08	*Hypotrachyna sinuosa* (Sm.) Hale	0.00
Sticta sp.1	0.06	Sp.6	0.00

Species with significant ($p < 0.05$) indval values are highlighted in grey and bold. Signif. codes: '***'=p<0.001, '**'=p<0.01,'*'=p<0.05, '.'=p<0.1. Species considered important components of each habitat type were given larger indval values and those seen to be more restricted to a certain habitat type were given higher significance values for that habitat type.

Table S5 cont. Indicator species analysis[79] (indval) values of epiphytic lichen species comparing deadwood and live tree habitat.

Deadwood indicator species	indval	Live tree indicator species	indval
Lecanora sp.	**0.06**	***Sticta* sp.3**	**0.00**
Usnea cornuta Körb.	0.05	*Cladonia merochlorophaea* Asahina	0.00
Psoroma hypnorum (Vahl) Gray	0.04	*Sticta sylvatica* (Huds.) Ach.	0.00
Leptogium mandonii P.M. Jørg.	0.04		
Sticta sp.2	0.03		
Sticta andensis (Nyl.) Trevis.	0.03		
Usnea fragilescens Hav. ex Lynge	0.03		
Milospium sp.	0.02		
Usnea esperantiana P. Clerc	0.02		
Leptogium laceroides B. de Lesd.	0.02		
Remototrachyna sp.	0.02		
Leptogium phyllocarpum (Pers.) Mont.	0.02		
Leptogium menziesii (Sm.) Mont.	0.02		
Pertusaria sp.	0.02		
Caloplaca sp.2	0.02		
Chaenotheca chlorella (Ach.) Müll.Arg.	0.02		
Dimerella lutea (Dicks.) Trevis.	0.02		
Collema fasciculare (L.) Weber ex F.H. Wigg.	0.02		
Bacidina sp.	0.02		
Stereocaulon glareosum (Savicz) H. Magn.	0.02		
Leprocaulon congestum (Nyl.) I.M. Lamb & A. Ward	0.01		
Sp.5	0.01		
Candelariella sp.1	0.01		
Sticta sublimbatoides Moncada & Lücking ined.	0.01		
Psorogalaena sp.	0.01		
Chaenothecopsis nivea (F. Wilson) Tibell	0.01		
Buellia proximata H. Magn.	0.01		
Lecidea sp.2	0.01		
Megalospora admixta (Nyl.) Sipman	0.01		
Megaspora verrucosa (Ach.) Hafellner & V. Wirth	0.01		
Sticta fuliginosa (Dicks.) Ach.	0.01		
Sticta maculofuliginosa Moncada & Lücking	0.01		
Sticta paramuna Moncada & Lücking ined.	0.01		
Sp.3	0.01		
Arthonia sp.2	0.01		
Hypotrachyna ensifolia (Kurok.) Hale	0.01		
Hypotrachyna sublaevigata (Nyl.) Hale	0.01		
Sp.4	0.01		
Porina sp.	0.01		

Species with significant (p <0.05) indval values are highlighted in grey and bold. Signif. codes: '***'=p<0.001, '**'=p<0.01,'*'=p<0.05, '.'=p<0.1. Species considered important components of each habitat type were given larger indval values and those seen to be more restricted to a certain habitat type were given higher significance values for that habitat type.

Table S5 cont. Indicator species analysis[79] (indval) values of epiphytic lichen species comparing deadwood and live tree habitat.

Deadwood indicator species	indval	Live tree indicator species	indval
Chaenothecopsis pusilla (Ach.) A.F.W. Schmidt	0.01		
Leptogium burgessii (L.) Mont.	0.01		
Cladonia cartilaginea Müll. Arg.	0.00		
Chaenothecopsis debilis (Sm.) Tibell	0.00		
Buellia disciformis (Fr.) Mudd	0.00		
Candelariella sp.2	0.00		
Chysothrix sp.	0.00		
Cladonia sp.	0.00		
Leptogium coralloideum (Meyen & Flot.) Vain.	0.00		
Leptogium cyanescens (Pers.) Körb.	0.00		
Pannaria andina P.M. Jørg. & Sipman	0.00		
Sp.8	0.00		
Sticta rhizinata Moncada & Lücking	0.00		
Usnea glabrata (Ach.) Vain.	0.00		
Usnea setulosa Motyka	0.00		
Usnea sp.2	0.00		

Species with significant (p <0.05) indval values are highlighted in grey and bold. Signif. codes: '***'=p<0.001, '**'=p<0.01,'*'=p<0.05, '.'=p<0.1. Species considered important components of each habitat type were given larger indval values and those seen to be more restricted to a certain habitat type were given higher significance values for that habitat type.

Table S6. Morphological traits, their corresponding scaled principal component loadings, eigenvalues and proportion of the data explained.

Trait	PC1 'low-growing herbs'	PC2 'tussock grasses'	PC3 'trees'	PC4 'geo-phytes'	PC5 'large creeping forbs'	PC6 'not large biennial herbs'	PC7 'short graminoid-s'	PC8 'not spiny or cushion forming'
Moderately hairy	**1.04**	-0.45	0.06	-0.24	0.57	-0.12	0.23	-0.01
Densely hairy	0.10	-0.08	-0.08	0.13	-0.16	-0.20	-0.24	0.32
Spiny or stinging	0.12	-0.03	-0.04	-0.11	-0.25	-0.21	**-1.00**	**-1.13**
Sclerophyllous	0.35	**1.04**	0.26	**-0.80**	0.21	-0.28	-0.07	-0.35
Tussocks	0.09	**1.30**	0.28	-0.67	0.45	-0.11	-0.14	0.06
Graminoid	0.41	**1.19**	0.17	-0.31	-0.60	-0.54	**0.70**	-0.21
Below-ground storage organs	**0.73**	0.37	0.03	**0.80**	-0.34	0.41	-0.67	0.54
Rhizomatous	0.50	0.68	-0.06	**1.45**	0.07	0.19	0.23	-0.23
Buds close to soil level	**1.41**	-0.12	0.00	0.57	-0.11	0.04	-0.26	-0.14
Stoloniferous	**1.26**	-0.46	0.11	-0.27	**0.77**	-0.09	0.27	-0.13
Creeping	**1.27**	-0.50	0.10	-0.30	**0.75**	-0.08	0.30	-0.14
Rosette	0.49	-0.02	-0.03	0.13	**-0.86**	0.04	**-0.83**	0.44
Cushion forming	0.10	0.00	-0.01	-0.14	-0.17	-0.07	**-0.82**	**-1.26**
Prostrate	**1.44**	-0.43	0.07	0.04	0.44	0.11	0.05	0.13
Veg. height over 300cm	-0.22	-0.28	**1.75**	0.24	-0.04	-0.14	-0.01	-0.01
Veg. height 50-300cm	0.12	1.49	0.27	-0.15	0.65	0.31	-0.35	0.26
Veg. height 15-50cm	-0.22	-0.06	-0.23	0.26	0.41	**-1.44**	-0.43	0.35
Veg. height 5-15cm	-0.01	0.36	-0.14	**0.82**	0.05	-0.31	0.75	-0.64
Veg. height 0-5cm	**1.53**	-0.01	0.08	-0.15	**-0.79**	-0.18	0.07	0.15
Reproduct. height over 300cm	-0.22	-0.28	**1.75**	0.24	-0.04	-0.14	-0.01	-0.01
Reproduct. height 50-300cm	0.16	**1.53**	0.25	0.07	0.63	0.29	-0.20	0.10
Reproduct. height 15-50cm	-0.18	0.10	-0.26	0.67	0.34	**-1.35**	-0.04	-0.02
Reproduct. height 5-15cm	0.41	0.46	0.03	-0.23	**-1.18**	-0.43	**0.71**	-0.04
Reproduct. height 0-5cm	**1.51**	-0.38	0.05	0.03	0.00	0.13	-0.36	0.13
Epiphyte	0.00	0.00	0.00	0.00	0.00	0.00	0.00	0.00
Nanophanerophyte	-0.13	0.35	0.05	-0.13	0.44	0.35	-0.26	0.28
Micro-mesophanerophyte	-0.21	-0.28	**1.75**	0.24	-0.04	-0.15	-0.01	-0.01
Geophyte	0.53	0.65	-0.07	**1.48**	0.05	0.22	0.17	-0.19
Chamaephyte	0.50	-0.20	0.03	-0.20	0.55	-0.19	0.03	-0.26
Hemicryptophyte	**1.11**	0.55	0.18	**-0.71**	-0.69	-0.48	-0.07	0.23
Biennial	-0.12	-0.04	-0.15	0.24	0.33	**-1.20**	-0.36	0.44
Therophyte	0.26	-0.26	-0.05	-0.08	0.13	0.11	0.42	-0.12
Eigenvalue	5.048	3.630	2.986	2.686	2.285	2.167	1.735	1.475
Proportion Explained	0.163	0.117	0.096	0.087	0.074	0.070	0.056	0.048
Cumulative Proportion	0.163	0.280	0.376	0.463	0.537	0.607	0.663	0.710

Numbers in bold indicate high loadings.

Table S7. Soil types of the soil profiles studied at sites 1 to 4.

Site	Mollic Leptosol	Phaeozem	Umbrisol
1 (n = 9)	2 (IG, IF)	4 (2 IF, 2 IG)	3 (AG)
2 (n = 12)	-	8 (2 IF, 1 IG, 3 AF, 2 AG)	4 (1 IF, 2 IG, 1 AG)
3 (n = 12)	-	5 (3 IF, 2 AF)	7 (3 IG, 1 AF, 3 AG)
4 (n = 6)	-	1 IF	5 (2 IG, 3 AG)
Total	2	18 (8 IF, 3 IG, 5 AF, 2 AG)	19 (1 IF, 7 IG, 1 AF, 10 AG)

IF=Inaccessible Forest, IG=Inaccessible Grassland, AF=Accessible Forest, AG=Accessible Grassland.
Soil types are determined according to IUSS Working Group WRB* and are presented on the level of Reference Soil Groups.

* “World reference base for soil resources 2014. International classification system for naming soils and creating legends for soil maps”, World Soil Resources Report (FAO, Rome, 2014).

Table S8. Values of aboveground tree carbon, Soil Organic Carbon (SOC), root carbon and total carbon per m^2 for inaccessible and accessible forest and grassland habitats.

Carbon stocks (kg m^{-2})	Value	Inaccessible forest	Inaccessible grassland	Accessible forest	Accessible grassland
tree carbon	min.	5.05		4.06	
	lower quartile	11.95		7.16	
	median	16.38		9.94	
	upper quartile	20.70		15.92	
	max.	33.47		22.92	
	mean	17.00		12.74	
SOC	min.	10.21	9.21	3.56	10.33
	lower quartile	10.56	14.68	7.73	12.81
	median	15.26	21.39	10.08	18.87
	upper quartile	18.37	26.07	10.89	22.45
	max.	20.07	32.07	10.89	26.23
	mean	15.02	20.77	12.39	18.11
root carbon	min.	0.40	0.30	0.21	0.05
	lower quartile	0.56	0.59	0.43	0.11
	median	1.03	0.90	0.63	0.44
	upper quartile	1.93	1.41	0.92	0.70
	max.	2.69	2.49	1.10	1.41
	mean	1.28	1.09	0.65	0.50
total carbon	min.	15.66	9.51	7.83	10.38
	lower quartile	23.07	15.27	15.31	12.92
	median	32.67	22.29	20.65	19.30
	upper quartile	41.01	27.48	27.74	23.15
	max.	56.22	34.56	34.91	27.64
	mean	33.31	21.86	25.78	18.60

Table S9. Potential (shaded in grey) and actual carbon stocks of the Cordillera Urubamba

carbon (C) stocks	area (km^2)	min. values of carbon stocks (Gg)	lower quartile values of carbon stocks (Gg)	median values of carbon stocks (Gg)	upper quartile values of carbon stocks (Gg)	max. values of carbon stocks (Gg)	mean carbon stocks (Gg)
**potential total tree C**	**54075.2**	**273.29**	**646.42**	**885.55**	**1119.60**	**1809.69**	**919.39**
**potential total root C**	**76889.7**	**28.32**	**43.64**	**76.48**	**136.83**	**202.17**	**94.41**
**potential total SOC**	**76889.7**	**762.23**	**905.83**	**1313.33**	**1588.34**	**1816.72**	**1286.14**
potential forest root C	54075.2	21.41	30.23	55.84	104.63	145.28	69.48
potential grassland root C	22814.5	6.92	13.41	20.65	32.20	56.89	24.93
potential forest SOC	54075.2	552.16	570.88	825.41	993.58	1085.13	812.32
potential grassland SOC	22814.5	210.07	334.95	487.92	594.76	731.59	473.81
potential forest C	54075.2	846.85	1247.53	1766.80	2217.80	3040.10	1801.20
potential grassland C	22814.5	216.98	348.36	508.56	626.97	788.48	498.74
potential total C	76889.7	1063.84	1595.89	2275.37	2844.77	3828.58	2299.94
**actual total tree C**	**6665.2**	**28.07**	**52.48**	**72.67**	**110.86**	**163.29**	**89.14**
**actual total root C**	**76889.7**	**5.35**	**11.21**	**35.49**	**56.65**	**108.63**	**40.04**
**actual total SOC**	**76889.7**	**755.03**	**954.38**	**1398.31**	**1657.86**	**1926.25**	**1357.83**
actual inaccessible forest tree C	995.2	5.03	11.90	16.30	20.61	33.31	16.92
actual inaccessible forest root C	995.2	0.39	0.56	1.03	1.93	2.67	1.28
actual inaccessible grassland root C	419.9	0.13	0.25	0.38	0.59	1.05	0.46
actual inaccessible forest SOC	995.2	10.16	10.51	15.19	18.29	19.97	14.95
actual inaccessible grassland SOC	419.9	3.87	6.16	8.98	10.95	13.46	8.72
actual accessible forest tree C	5670.0	23.04	40.58	56.37	90.26	129.98	72.21
actual accessible forest root C	5670.0	1.21	2.41	3.56	5.24	6.22	3.70
actual accessible grassland root C	69804.6	3.62	8.00	30.53	48.89	98.69	34.60
actual accessible forest SOC	5670.0	20.16	43.81	57.14	61.76	61.76	70.24
actual accessible grassland SOC	69804.6	720.84	893.89	1316.99	1566.87	1831.05	1263.92
total actual inaccessible forest C	995.2	15.59	22.96	32.52	40.82	55.95	33.15
total actual inaccessible grassland C	419.9	3.99	6.41	9.36	11.54	14.51	9.18
total actual accessible forest C	5670.0	44.41	86.80	117.07	157.26	197.96	146.15
total actual accessible grassland C	69804.6	724.46	901.89	1347.52	1615.75	1929.74	1298.52
actual total	76889.7	788.46	1018.07	1506.47	1825.37	2198.17	1487.00

Estimated values of potential (shaded in grey) and actual carbon stocks in the high elevation area mapped of the Cordillera Urubamba (132,660 km^2; Fig. 1a, 2a). Values estimated using carbon stock values from inaccessible and accessible forest and grassland (Table S8) extrapolated over the areas calculated using landscape mapping (Table S1). ‘tree’ refers to the aboveground carbon of trees. Values for potential and current overall tree, root and SOC carbon stocks are in bold and shown graphically in Fig. 5d.

Table S10. Estimated values of current carbon stocks per m^2 for inaccessible and accessible landscapes given the proportions of forest and grassland vegetation type calculated from landscape mapping (see Table S1) and the values of carbon per habitat type in Table S8.

Habitat	min. values of carbon stocks (kg m^{-2})	lower quartile values of carbon stocks (kg m^{-2})	median values of carbon stocks (kg m^{-2})	upper quartile values of carbon stocks (kg m^{-2})	max. values of carbon stocks (kg m^{-2})	mean carbon stocks (kg m^{-2})
Inaccessible	13.84	20.76	29.59	37.00	49.80	29.91
Accessible	10.19	13.10	19.41	23.49	28.19	19.14

Table S11. Mean climate values of the Cordillera Urubamba and Vilcabamba averaging all datalogger sources.

	Absolute minimum temperature (øC)	Absolute maximum temperature (øC)	Mean daily minimum temperature (øC)	Mean daily maximum temperature (øC)	Mean daily temperature (øC)	Mean daily temperature variation	Absolute max daily temperature variation	Days yr^{-1} where max temperature doesn't reach 5øC	Days yr^{-1} where temperature dropped equal or below 0øC	Absolute minimum relative humidity (%)	Mean daily relative humidity (%)	Days yr^{-1} where relative humidity passed 90%	Days yr^{-1} with fog occurrence (relative humidity passed 99%)
Urubamba	-3.9	27.3	0.6	11.7	4.6	11.1	27.9	34.0	167.8	20.3	84.7	261.3	180.9
Vilcabamba	-6.4	17.6	1.2	8.7	4.1	7.5	19.2	25.1	88.1	24.1	90.7	299.3	207.3

Urubamba values based on 12 dataloggers placed at sites 1–4 with recordings made between October 2010–July 2012. Vilcabamba values based on 7 dataloggers placed at sites 5, 6 and 8 with recordings made between May 2012–April 2013. Site 5, although belonging to the Cordillera Urubamba, had a climate more similar to that of the Cordillera Vilcabamba with a correspondingly similar vegetation, and so is treated here as such.

Table S12. LMM analysis results of the differences in climate properties between inaccessible and accessible sites in forest habitats.

Climate property (transformation, if any, in brackets)	Effect	Estimate	Std. Error	df	t value	Pr(>|t|)
Mean daily temperature (log10)	(Intercept)	1.38653	0.06112	11.771	22.684	<0.001
	Human impact	0.14484	0.08464	10.829	1.711	0.115
Mean daily minimum temperature	(Intercept)	0.7128	0.3966	12.505	1.797	0.0965
	Human impact	0.1539	0.4745	11.015	0.324	0.7518
Mean daily maximum temperature	(Intercept)	9.832	1.761	10.152	5.582	<0.001
	Human impact	1.938	2.276	5.054	0.851	0.433009
Absolute minimum temperature	(Intercept)	-4.028	1.525	17	-2.641	0.0171
	Human impact	-1.447	2.10E+00	17	-0.688	0.5006
Absolute maximum temperature (log10)	(Intercept)	3.0305	0.1245	13.04	24.351	<0.001
	Human impact	0.1302	0.1601	10.299	0.813	0.435
Mean daily temperature variation (log10)	(Intercept)	2.1155	0.1482	12.37	14.277	<0.001
	Human impact	0.1434	0.189	9.627	0.759	0.466
Absolute max. daily temperature variation (log10)	(Intercept)	3.033	0.1446	17	20.98	<0.001
	Human impact	0.1565	0.1993	17	0.786	0.443
Days yr where max. temperature does not reach 5øC (log10)	(Intercept)	3.0486	0.4126	13.742	7.389	<0.001
	Human impact	-0.5616	0.5633	7.15	-0.997	0.351
Days yr where temperature dropped equal or below 0øC	(Intercept)	152.78	32.63	17	4.683	<0.001
	Human impact	-27.28	44.97	17	-0.607	0.552161
Mean daily relative humidity	(Intercept)	88.027	2.155	10.234	40.85	<0.001
	Human impact	-2.358	2.204	6.844	-1.07	0.321
Days yr where relative humidity passed 90%	(Intercept)	280.67	19.32	17	14.525	<0.001
	Human impact	-10.17	26.64	17	-0.382	0.707
Days yr with fog occurrence (relative humidity passed 99%)	(Intercept)	203	16	17	12.686	<0.001
	Human impact	-23.5	22.06	17	-1.065	0.302
Absolute minimum relative humidity (log10)	(Intercept)	3.0077	0.2484	12.689	12.109	<0.001
	Human impact	-0.3797	0.324	6.117	-1.172	0.285

If values were transformed prior to analysis, the type of transformation is shown in brackets alongside the name of the property. Our models used the fixed effect, human impact, and two sets of random effects that crossed the fixed effects with sites 1–6, 8 and the covariate elevation. The climate properties were calculated from temperature and relative humidity data collected from 19 data sources; 9 from inaccessible habitats and 10 from accessible habitats across the seven sites (See Table S11).

Table S13. GLMM analysis results of the effect of human impact, vegetation type and the interaction between human impact and vegetation type on different vegetation type structure properties.

Vegetation type structure property	Effect	Estimate	Std. Error	z value	Pr(>|z|)
Proportion of standing deadwood trees per 10x10m^2 plot [logit] (Gaussian GLMM with identity link function and zero inflation being taken into account)	(Intercept)	3.547	0.158	-22.5	<0.001
	Human impact	-1.477	0.197	-7.48	<0.001
Average height of forb communities per 2x2m^2 plot (Gamma GLMM with log link function)	(Intercept)	3.3186	0.0806	41.15	<0.001
	Human impact	-1.3264	0.075	-17.68	<0.001
	Vegetation type	0.2849	0.0818	3.48	<0.001
	Interaction	-0.6249	0.1189	-5.25	<0.001
Maximum height of forb communities per 2x2m^2 plot (Gamma GLMM with log link function)	(Intercept)	4.3573	0.0577	75.54	<0.001
	Human impact	-0.7631	0.0541	-14.1	<0.001
	Vegetation type	0.0702	0.0596	1.18	0.239
	Interaction	-0.1432	0.0851	-1.68	0.093
Cover of bare soil per 2x2m^2 plot [logit] (Gaussian GLMM with identity link function and zero inflation being taken into account)	(Intercept)	-3.3246	0.1213	-27.4	<0.001
	Human impact	1.3039	0.0987	13.21	<0.001
	Vegetation type	0.1842	0.1048	1.76	0.079
	Interaction	-0.2694	0.15	-1.8	0.073
Cover of litter per 2x2m^2 plot [logit] (Gaussian GLMM with identity link function)	(Intercept)	0.0914	0.2165	0.42	0.67
	Human impact	-2.2581	0.125	-18.06	<0.001
	Vegetation type	0.1975	0.138	1.43	0.15
	Interaction	-0.8766	0.1966	-4.46	<0.001
Percentage grazing per 2x2m^2 plot [logit] (Gaussian GLMM with identity link function and zero inflation being taken into account)	(Intercept)	-3.3130	0.1993	-16.63	<0.001
	Human impact	2.2324	0.0848	26.32	<0.001
	Vegetation type	0.09526	0.0936	1.02	0.31
	Interaction	-0.00129	0.1334	-0.01	0.99

If values were transformed prior to analysis, the type of transformation is shown in square brackets alongside the name of the property. The exponential family and link function of the GLMM is written in parentheses alongside the name of the property. Our models used fixed effects (human impact + vegetation type + human impact × vegetation type) and random effects. for ‘Proportion of standing deadwood trees per 10x10m^2 plot’, the random effects ‘tree species’, ‘Cordillera’ and ‘elevation’ were used. To treat elevation as a random effect, elevational values 4211–4812 m a.s.l. were converted into a factor with 20 levels with each level referring to an elevational difference of 30.05 m. The random effect for all other vegetation type structure properties was the factor ‘site’ with 8 levels corresponding to sites 1-8 across the Cordilleras Urubamba and Vilcabamba.

Table S14. LMM analysis results of the effect of human impact on forest structure properties.

Vegetation type structure property	Effect	Estimate	Std. Error	df	t value	Pr(>|t|)
Total aboveground biomass of trees per 10x10m^2 plot (log10)	(Intercept)	7.6985	0.1944	1.24	39.609	0.00716
	Human impact	0.434	0.1483	46	2.926	0.00531
Average aboveground biomass of trees per 10x10m^2 plot	(Intercept)	100.187	3.76	0.71	26.643	0.0611
	Human impact	5.249	2.209	40.67	2.376	0.0223
Max. aboveground biomass of trees per 10x10m^2 plot	(Intercept)	111.893	3.844	0.86	29.108	0.0347
	Human impact	9.396	1.776	43.57	5.292	<0.001
Stand basal area (log10)	(Intercept)	5.8983	0.3345	1.09	17.633	0.0288
	Human impact	1.0061	0.1621	45.62	6.207	<0.001
Mean dbh of trees per 10x10m^2 plot (log10)	(Intercept)	2.5791	0.2694	1.09	9.573	0.0551
	Human impact	0.2543	0.1192	42.22	2.134	0.0387
Mean height of trees per 10x10m^2 plot (log10)	(Intercept)	1.5383	0.13768	4.19	11.173	<0.001
	Human impact	0.31495	0.09821	42.29	3.207	0.002556
Max. height of trees per 10x10m^2 plot (log10)	(Intercept)	2.7461	0.2114	3.62	12.987	<0.001
	Human impact	0.7302	0.1241	41.16	5.883	<0.001

If values were transformed prior to analysis, the type of transformation is shown in brackets alongside the name of the property. Our models used the fixed effect, human impact, and random effects (tree species, Cordillera, elevation).

Table S15. GLMM analysis results on the effect of human impact, vegetation type and the interaction between human impact and vegetation type on different biodiversity properties.

Ecosystem property	Effect	Estimate	Std. Error	z value	Pr(>|z|)
Vascular plant species richness per 2x2m^2 plot (Negative binomial GLMM with log link function to account for the heterogeneity of variance)	(Intercept)	2.1944	0.0571	38.45	<0.001
	Human impact	0.8904	0.0451	1.97E+01	<0.001
	Vegetation type	0.0407	0.056	0.73	0.47
	Interaction	0.0641	0.0721	0.89	0.37
Cover of undescribed spp. per 2x2m^2 plot [logit] (Gaussian GLMM with identity link function and zero inflation being taken into account)	(Intercept)	-0.445	0.334	-1.34	0.182
	Human impact	-2.868	0.21	-1.37E+01	<0.001
	Vegetation type	-0.469	0.232	-2.02	0.043
	Interaction	0.456	0.33	1.38	0.167
Proportion of undescribed species per 2x2m^2 plot [logit] (Gaussian GLMM with identity link function and zero inflation being taken into account)	(Intercept)	20.69	1.75	11.8	<0.001
	Human impact	-17.69	1.78	-9.94	<0.001
	Vegetation type	-2.47	1.71	-1.45	0.15
	Interaction	2.5	2.78	0.9	0.37
Cover of introduced species per 2x2m^2 plot [logit] (Gaussian GLMM with identity link function and zero inflation being taken into account)	(Intercept)	-3.6649	0.0658	-55.69	<0.001
	Human impact	0.4478	0.0429	10.44	<0.001
	Vegetation type	0.0197	0.0465	0.42	0.6722
	Interaction	-0.2055	0.0675	-3.04	0.0023
Proportion of introduced species per 2x2m^2 plot [logit] (Gaussian GLMM with identity link function and zero inflation being taken into account)	(Intercept)	-3.6709	0.1063	-34.53	<0.001
	Human impact	0.8355	0.0601	13.91	<0.001
	Vegetation type	0.0296	0.0663	0.45	0.65511
	Interaction	-0.3538	0.0945	-3.74	<0.001
Average latitudinal range size of all vascular plant species per 2x2m^2 plot (Gamma GLMM with log link function)	(Intercept)	6.8052	0.0583	116.8	<0.001
	Human impact	0.7798	0.0481	16.22	<0.001
	Vegetation type	0.4188	0.053	7.9	<0.001
	Interaction	-0.308	0.0758	-4.06	<0.001
Average latitudinal range size of vascular plant species per 2x2m^2 plot excluding introduced species (Gamma GLMM with log link function)	(Intercept)	6.8053	0.0515	132.08	<0.001
	Human impact	0.6427	0.0475	13.54	<0.001
	Vegetation type	0.4098	0.0525	7.81	<0.001
	Interaction	-0.2133	0.0750	-2.84	0.0045
Epiphytic lichen species richness per 600cm^2 plot (Negative binomial GLMM with log link function to account for the heterogeneity of variance)	(Intercept)	1.9965	0.1899	10.51	<0.001
	Human impact	-0.6461	0.0698	-9.26	<0.001

If values were transformed prior to analysis, the type of transformation is shown in square brackets alongside the name of the property. The exponential family and link function of the GLMM is written in parentheses alongside the name of the property. In all properties apart from ‘Epiphytic lichen species richness per 600cm^2 plot’, our models used fixed effects (human impact + vegetation type + human impact × vegetation type) and one set of random effects that crossed these fixed effects with sites 1–8 across the Cordilleras Urubamba and Vilcabamba. For property ‘Epiphytic lichen species richness per 600cm^2 plot’, the fixed effect was ‘substrate type’, with the two levels ‘deadwood’ and ‘live’, while the factor ‘site’, with four levels corresponding to the sites 1–4 of the Cordillera Urubamba, was treated as a random effect.

Table S16. LMM analysis results of the effect of human impact, vegetation type and the interaction between human impact and vegetation type on plant trait syndromes represented by principal components.

Principal component	Effect	Estimate	Std. Error	df	t value	Pr(>|t|)
PC1 'low-growing herbs'	(Intercept)	-0.01721	0.04361	9.1	-0.394	0.702
	Human impact	0.55008	0.02978	465.5	18.472	<0.001
	Vegetation type	0.29292	0.03294	465	8.892	<0.001
	Interaction	0.01966	0.04693	464.6	0.419	0.675
PC2 'tussock grasses'	(Intercept)	0.66583	0.07378	10.9	9.025	<0.001
	Human impact	-0.62479	0.05387	466.3	-11.597	<0.001
	Vegetation type	0.27328	0.0596	465.9	4.585	<0.001
	Interaction	-0.29732	0.08491	465.6	-3.502	<0.001
PC3 'trees'	(Intercept)	1.26177	0.07891	9.7	15.989	<0.001
	Human impact	-0.74503	0.05051	465.3	-14.749	<0.001
	Vegetation type	-0.9244	0.05588	464.8	-16.544	<0.001
	Interaction	0.47708	0.07959	464.4	5.994	<0.001
PC4 'geophytes'	(Intercept)	0.31543	0.09497	8.5	3.321	0.00959
	Human impact	-0.01624	0.04293	463	-0.378	0.7054
	Vegetation type	-0.34628	0.04747	462.6	-7.295	<0.001
	Interaction	0.13247	0.0676	462.1	1.96	0.05065
PC5 'large creeping forbs'	(Intercept)	0.32513	0.06338	10.1	5.13	<0.001
	Human impact	-0.55231	0.03913	465.2	-14.113	<0.001
	Vegetation type	-0.17307	0.04329	464.8	-3.998	<0.001
	Interaction	-0.03488	0.06166	464.3	-0.566	0.571909
PC6 'not large biennial herbs'	(Intercept)	-0.33607	0.04806	13.2	-6.993	<0.001
	Human impact	0.17684	0.0413	467.4	4.282	<0.001
	Vegetation type	-0.20814	0.0457	467	-4.554	<0.001
	Interaction	0.26425	0.06511	467.2	4.059	<0.001
PC7 'short graminoids'	(Intercept)	-0.03736	0.072	8.6	-0.519	0.617
	Human impact	0.1981	0.02771	462.3	7.149	<0.001
	Vegetation type	-0.01022	0.03064	462.1	-0.334	0.739
	Interaction	-0.01131	0.04363	461.7	-0.259	0.796
PC8 'not spiny or cushion forming'	(Intercept)	0.04601	0.04777	8.7	0.963	0.361416
	Human impact	-0.08383	0.02276	463.3	-3.684	<0.001
	Vegetation type	0.05182	0.02517	462.9	2.059	0.040021
	Interaction	-0.0577	0.03584	462.4	-1.61	0.108058

Our models used fixed effects (human impact + vegetation type + human impact × vegetation type) and one set of random effects that crossed these fixed effects with sites 1-8 across the Cordilleras Urubamba and Vilcabamba.

Table S17. LMM analysis results of the effect of human impact, vegetation type and the interaction between human impact and vegetation type on different soil properties.

Soil property (transformation in brackets)	Effect	Estimate	Std. Error	df	t value	Pr(>|t|)
CEC (log10)	(Intercept)	7.87834	0.14757	16.77	53.387	<0.001
	Human impact	-0.35523	0.22052	34.38	-1.611	0.116
	Vegetation type	-0.28446	0.18379	32.91	-1.548	0.131
	Interaction	0.03694	0.28217	34	0.131	0.897
pH	(Intercept)	8.20E-05	1.98E-05	9.06E+00	4.144	0.00247
	Human impact	-1.54E-05	2.52E-05	3.34E+01	-0.612	0.54493
	Vegetation type	1.10E-05	2.08E-05	3.24E+01	0.527	0.60177
	Interaction	6.05E-06	3.22E-05	3.31E+01	0.188	0.85208
Mn (log10)	(Intercept)	1.60962	0.13683	5.9	11.763	<0.001
	Human impact	0.08187	0.14421	32.73	0.568	0.57408
	Vegetation type	-0.0176	0.11858	32.12	-0.148	0.88294
	Interaction	-0.53825	0.18379	32.57	-2.929	0.00617
Nt (sqrt)	(Intercept)	33.8743	3.6688	4.58	9.233	<0.001
	Human impact	-3.2857	3.2508	32.37	-1.011	0.319641
	Vegetation type	5.6965	2.6681	31.97	2.135	0.040519
	Interaction	-0.5064	4.1409	32.26	-0.122	0.903434
Ca (log10)	(Intercept)	7.1469	0.3521	9.47	20.297	<0.001
	Human impact	-0.214	0.4668	33.41	-0.458	0.64966
	Vegetation type	-1.0812	0.386	32.17	-2.801	0.00855
	Interaction	-0.711	0.5959	33.08	-1.193	0.24127
Mg (sqrt)	(Intercept)	22.072	2.762	12.09	7.991	<0.001
	Human impact	-7.525	4.008	33.86	-1.878	0.0691
	Vegetation type	-10.88	3.332	32.02	-3.266	0.0026
	Interaction	5.493	5.124	33.38	1.072	0.2915
K (sqrt)	(Intercept)	8.802	0.7194	5.58	12.235	<0.001
	Human impact	-0.527	0.7075	32.72	-0.745	0.462
	Vegetation type	-0.508	0.5813	32.23	-0.874	0.389
	Interaction	-1.1077	0.9015	32.58	-1.229	0.228
Na (sqrt)	(Intercept)	4.0365	0.4021	8.4	10.038	<0.001
	Human impact	-1.2432	0.509	33.22	-2.442	0.0201
	Vegetation type	-0.6499	0.4203	32.16	-1.546	0.1318
	Interaction	0.7278	0.6495	32.93	1.121	0.2705
Al	(Intercept)	537.53	188.89	8.81	2.846	0.0196
	Human impact	-99.28	249.78	33.26	-0.397	0.6936
	Vegetation type	548.4	206.55	31.94	2.655	0.0123
	Interaction	-87.71	318.85	32.9	-0.275	0.785

If values were transformed prior to analysis, the type of transformation is shown in brackets alongside the name of the property. Our models used fixed effects (human impact + vegetation type + human impact × vegetation type) and one set of random effects that crossed the fixed effects with sites 1–4. Absolute pH values were not used, but, instead, we used the actual proton (H+) concentration as this is metric and rationally scaled.

Table S17 cont. LMM analysis results of the effect of human impact, vegetation type and the interaction between human impact and vegetation type on different soil properties.

Soil property (transformation in brackets)	Effect	Estimate	Std. Error	df	t value	Pr(>|t|)
Fe (log10)	(Intercept)	0.49644	0.14045	15.19	3.534	0.00295
	Human impact	-0.08065	0.21171	34.28	-0.381	0.70561
	Vegetation type	-0.0729	0.17662	32.39	-0.413	0.6825
	Interaction	-0.05628	0.27098	33.8	-0.208	0.83671
Mn (log10)	(Intercept)	1.60962	0.13683	5.9	11.763	<0.001
	Human impact	0.08187	0.14421	32.73	0.568	0.57408
	Vegetation type	-0.0176	0.11858	32.12	-0.148	0.88294
	Interaction	-0.53825	0.18379	32.57	-2.929	0.00617
H (sqrt)	(Intercept)	5.4836	0.9644	7.42	5.686	<0.001
	Human impact	-1.115	1.1472	33.05	-0.972	0.338129
	Vegetation type	0.2673	0.9455	32.2	0.283	0.779164
	Interaction	-0.3398	1.463	32.82	-0.232	0.817782
bases (log10)	(Intercept)	7.5303	0.2972	10.4	25.337	<0.001
	Human impact	-0.3467	0.4055	33.58	-0.855	0.39856
	Vegetation type	-0.9937	0.3358	32.22	-2.959	0.00574
	Interaction	-0.467	0.5178	33.21	-0.902	0.37364
C:N ratio	(Intercept)	14.2585	0.9443	4.42	15.099	<0.001
	Human impact	0.1484	0.7743	32.39	0.192	0.849
	Vegetation type	-0.8538	0.6351	32.06	-1.344	0.188
	Interaction	0.4362	0.9861	32.3	0.442	0.661
Rock content	(Intercept)	0.49397	0.06557	11.14	7.533	<0.001
	Human impact	0.02244	0.08844	33.71	0.254	0.801
	Vegetation type	-0.0269	0.0732	32.57	-0.367	0.716
	Interaction	-0.13398	0.11293	33.41	-1.186	0.244
Root biomass (sqrt)	(Intercept)	46.175	6.207	5.13	7.439	<0.001
	Human impact	-11.56	6.156	3.25E+01	-1.878	0.069385
	Vegetation type	-2.525	5.058	31.94	-0.499	0.621064
	Interaction	-3.578	7.844	32.33	-0.456	0.651296
Bulk density (log10)	(Intercept)	-0.52422	0.17122	4.52	-3.062	0.032
	Human impact	0.15506	0.1448	32.41	1.071	0.292
	Vegetation type	0.06768	0.1188	32.06	0.57	0.573
	Interaction	-0.07991	0.18443	32.31	-0.433	0.668
Soil mass	(Intercept)	265.123	50.216	7.24	5.28	0.00103
	Human impact	-6.769	59.803	32.99	-0.113	0.91057
	Vegetation type	1.416	49.289	32.11	0.029	0.97726
	Interaction	-3.845	76.267	32.75	-0.05	0.9601

If values were transformed prior to analysis, the type of transformation is shown in brackets alongside the name of the property. Our models used fixed effects (human impact + vegetation type + human impact × vegetation type) and one set of random effects that crossed the fixed effects with sites 1–4. Absolute pH values were not used, but, instead, we used the actual proton (H+) concentration as this is metric and rationally scaled.

Table S17 cont. LMM analysis results of the effect of human impact, vegetation type and the interaction between human impact and vegetation type on different soil properties.

Soil property (transformation in brackets)	Effect	Estimate	Std. Error	df	t value	Pr(>|t|)
Soil depth	(Intercept)	60.236	8.792	5.3	6.852	<0.001
	Human impact	-16.282	8.573	32.6	-1.899	0.0664
	Vegetation type	-2.856	7.043	32.1	-0.406	0.6878
	Interaction	2.236	10.923	32.47	0.205	0.8391

If values were transformed prior to analysis, the type of transformation is shown in brackets alongside the name of the property. Our models used fixed effects (human impact + vegetation type + human impact × vegetation type) and one set of random effects that crossed the fixed effects with sites 1–4. Absolute pH values were not used, but, instead, we used the actual proton (H+) concentration as this is metric and rationally scaled.

Table S18. LMM analysis results of the effect of human impact, vegetation type and the interaction between human impact and vegetation type on different carbon stocks.

Carbon stock (transformation in brackets)	Effect	Estimate	Std. Error	df	t value	Pr(>|t|)
Soil Organic Carbon: SOC	(Intercept)	15958.1	2746.12	6.05	5.811	0.0011
	Human impact	-1523.72	3077.28	32.67	-0.495	0.6238
	Vegetation type	5235.45	2532.98	31.89	2.067	0.0469
	Interaction	-1563.3	3922.99	32.46	-0.398	0.6929
Root Carbon (sqrt)	(Intercept)	1.03251	0.13879	5.13	7.439	<0.001
	Human impact	-0.2585	0.13765	32.48	-1.878	0.069385
	Vegetation type	-0.05646	0.1131	31.94	-0.499	0.621062
	Interaction	-0.08001	0.17539	32.33	-0.456	0.651299
Tree Carbon (log10)	(Intercept)	2.4002	0.1944	1.24	12.349	0.03018
	Human impact	0.434	0.1483	46	2.926	0.00531

If values were transformed prior to analysis, the type of transformation is shown in brackets alongside the name of the property. Our models used fixed effects (human impact + vegetation type + human impact × vegetation type) and random effects (sites 1–4 of the Cordillera Urubamba for ‘SOC’ and ‘Root Carbon’; tree species, Cordillera, elevation for ‘Tree Carbon’).
